# Research on false information clarification mechanism among government, opinion leaders, and Internet users — Based on differential game theory

**DOI:** 10.3389/fpsyg.2022.991337

**Published:** 2022-11-02

**Authors:** Bowen Li, Hua Li, Qiubai Sun, Rongjian Lv

**Affiliations:** ^1^School of Electronic and Information Engineering, University of Science and Technology Liaoning, Anshan, China; ^2^School of Business Administration, University of Science and Technology Liaoning, Anshan, China

**Keywords:** differential game, false information classification system, major emergencies, optimal control, opinion leaders

## Abstract

This article considers the government, opinion leaders, and Internet users to be a system for correcting false information, and it considers the problem of correcting false information that arises in the aftermath of major emergencies. We use optimal control theory and differential game theory to construct differential game models of decentralized decision-making, centralized decision-making, and subsidized decision-making. The solutions to these models and their numerical simulations show that the government, opinion leaders, and Internet users exercise cost-subsidized decision-making instead of decentralized decision-making. The equilibrium strategies, local optimal benefits, and overall optimal benefits of the system achieve Pareto improvement. Given the goal of maximizing the benefits to the system under centralized decision-making, the equilibrium results are Pareto-optimal. The research here provides a theoretical basis for dealing with the mechanism of correcting false information arising from major emergencies, and our conclusions provide methodological support for the government to effectively deal with such scenarios.

## Introduction

With rapid advances in Internet technologies, users from different parts of the world can obtain a variety of information related to major emergencies arising anywhere on the globe. However, due to the contingency and seriousness of major emergencies (Li et al., 2022), the government cannot immediately grasp the entire situation to quickly conduct the corresponding investigations after an emergency and provide the requisite information to the public. This leads to a period in which accurate information regarding a major emergency is unavailable on social media platforms soon after it occurs. During this period, Internet users with nefarious purposes can spread false information related to the emergency. If such information gains traction on the Internet and is widely disseminated, this can make it difficult for the relevant government departments to deal with the emergency, and can also threaten social stability. It is thus important to mitigate the public impact of the propagation of false information in the aftermath of major emergencies by constructing a system to correct such information.

A number of researchers have investigated the adverse effects of major emergencies. Hong et al. (2022) used complex networks to study the transmission of panic among Internet users after major emergencies. McElroy et al. (2020) claimed that major emergencies have a serious mental health impact on the public, and Akalu et al. (2021) claimed that such emergencies affect people physically as well as psychologically. Liu (2022) has argued that the release of correct information by opinion leaders can reduce panic among Internet users after major emergencies. Some scholars have also examined the interaction between actors after major emergencies. Wu et al. (2019) used the “Guangdong Maoming PX incident” in 2014 as an example to discuss the informational interaction between the government and the public in its aftermath. Cao et al. (2019) studied the influence of different types of extreme preference groups on the decision-making behavior of the public after the occurrence of emergencies. By taking the COVID-19 pandemic as an example, Xu et al. (2021) investigated behaviors related to pandemic prevention and control by the government, private enterprises, and the public based on the evolutionary game model. Din et al. (2022) also considered COVID-19 to examine the behavior of various actors in the food supply chain. Kang et al. (2022) studied helpful behaviors in this context between e-commerce platforms and their affiliated enterprises to assess the benefits of different rescue strategies after major emergencies.

Some scholars have studied the role of opinion leaders (Aleahmad et al., 2016; Bamakan et al., 2019; Jain and Katarya, 2019) in the context of the dissemination and correction of false information. This article defines opinion leaders as people who can influence and shape the opinions of others through their own words and actions. Hosseini and Zandvakili (2022) claimed that the degree of public trust in opinion leaders determines whether false information can spread. Yu et al. (2021) studied the dissemination of false information by constructing a model for it and proposed that the convenience of online networks enables the quick propagation of false information. Ozturk et al. (2015) argued that the emergence of social networking platforms has greatly enhanced users’ access to information but has also promoted the spread of false information. Bouanan et al. (2016) claimed that people’s decision-making behaviors are influenced by the information released by other individuals with whom they interact. Guess et al. (2019) argued that false information can change the behavior of the public to a certain extent, and Pal and Banerjee (2021) concluded that the content of false information is more attractive to Internet users than real information. Buchanan and Benson (2019) believe that both opinion leaders and ordinary Internet users can help spread false information, but the false information spread by the former has a wider range of influence. Bordia et al. (2005) claimed that credible opinion leaders can clarify false information by releasing correct information, and Vosoughi et al. (2018) suggested that it has a stronger capability of transmission and range of radiation than real information such that it can attract the attention of Internet users. Liao and Wang (2021) argued that the timely release of the latest reports on major emergencies to correct false information can greatly reduce its adverse effects. Agarwal et al. (2022) claimed that the government and opinion leaders should use social networking platforms to release correct information in a timely manner to reduce the impact of false information.

Researchers initially combined game theory with optimal control theory (Zhang et al., 2011) to study the problem of optimal control in the context of war (Perelman et al., 2011) and proposed differential game theory. With continued developments in differential game theory, it is now used as a theoretical tool for analyzing decision-making behavior (Fibich et al., 2003; Garcia-Meza, 2021). Biancardi et al. (2022) studied public water regulation and its use by water authorities and farmers in different decision-making situations. Prasad and Sethi (2004) used the differential game model to study the optimal investment in advertising, and Machowska et al. (2022) applied differential game theory to study the intentions of advertisers. Shchelchkov (2022) used differential game theory to study the problem of tracking and escaping among individuals. The differential game, as used in preceding studies, can be defined as an optimal control process in which different subjects interact with one another. Few studies have used differential game theory to examine the correction of false information after major emergencies. In this article, the authors regard the government, opinion leaders, and Internet users as a system in the aftermath of major emergencies, and they investigate the correction of false information by using optimal control theory and differential game theory. The following steps are employed in differential game theory. (1) We confirm the dynamic equation that relates the cost of the behavior of each subject in terms of effort with the total amount of real information. (2) We clarify the decision-making problems faced by each subject in different decision-making situations. (3) In light of the decision-making problem, we provide the optimal equilibrium strategy, the optimal benefit, the optimal trajectory of the total amount of real information, and the optimal benefits of the system to correct false information. (4) The equilibrium results are theoretically examined.

We take the government, opinion leaders, and Internet users as the objects of research to reduce the adverse public impact of false information spread in the aftermath of major emergencies based on differential game theory and optimal control theory.

First, we construct differential game models under decentralized decision-making, centralized decision-making, and cost-subsidized decision-making. Second, we solve the models to obtain their equilibrium results under different decision-making scenarios. Finally, we compare and analyze these results, and conduct numerical simulations on MATLAB 2017b to verify the theoretical analysis. The research here provides a theoretical basis for dealing with the mechanism of correcting false information after major emergencies. Our conclusions provide methodological support for the government to deal with such scenarios.

## Problem description and basic assumptions

### Problem description

To avoid panic among online users due to the dissemination of false information in the aftermath of major emergencies, we construct a system to correct such information that is composed of the government (G), opinion leaders (L), and Internet users (U). When a major emergency occurs, opinion leaders can choose to release the correct information, obtained through investigations and evidence collection, to Internet users on social platforms. Online users are eager for correct information. By paying attention to and disseminating correct information released by opinion leaders, a greater number of Internet users can participate in discussions of this information to widen access to it. The government can publish the correct information and can incentivize its propagation by opinion leaders and Internet users by establishing appropriate reward mechanisms for them.

### Model assumptions

Assumption 1: The respective efforts by the government, opinion leaders, and Internet users at time t can be, respectively, represented as follows:*E*_*G*_(*t*), *E*_*L*_(*t*), and*E*_*U*_(*t*).

Assumption 2: The costs to the government, opinion leaders, and Internet users are related to their own efforts, and constitute a convex function. The costs of efforts by the three entities at time t are as follows:


(1) $$\left\{ \begin{array}{c} C_{G}\,\left(t\right)=\frac{1}{2}\,\mu_{G}\,E_{G}^{2}\,\left(t\right)\\ C_{L}\,\left(t\right)=\frac{1}{2}\,\mu_{L}\,E_{L}^{2}\,\left(t\right)\\ C_{U}\,\left(t\right)=\frac{1}{2}\,\mu_{U}\,E_{U}^{2}\,\left(t\right) \end{array}\right.$$

where*C*_*G*_(*t*), *C*_*L*_(*t*), and *C*_*U*_(*t*) are the costs of efforts by the government, opinion leaders, and Internet users at time t, respectively, andμ_*G*_ > 0, μ_*L*_ > 0, and μ_*U*_ > 0 are the respective coefficients of the costs of their effort.

Assumption 3: The total amount of real information on the social networking platform is affected by the efforts of the government, opinion leaders, and Internet users. Considering the time lag of information, some real information has not been widely disseminated at a given time and thus cannot be used to correct false information. Therefore, we assume that the process of change in the total amount of real information on the social platform over time is:


(2) $$\left\{ \begin{array}{c} \dot{R}\,\left(t\right)=\alpha_{G}\,E_{G}\,\left(t\right)+\alpha_{L}\,E_{L}\,\left(t\right)+\alpha_{U}\,E_{U}\,\left(t\right)-\delta \,R\,\left(t\right)\\ R\,\left(0\right)=R_{0}\geq 0 \end{array}\right.$$

In the above, *R(t)* is the total amount of real information at time t, andα_*G*_ > 0, α_*L*_ > 0, and α_*U*_ > 0 are the impacts of efforts by the government, opinion leaders, and Internet users on the amount of real information. δ > 0is the coefficient of the natural dissipation of real information.

Assumption 4: The government, opinion leaders, and Internet users all benefit from the traffic generated by social networking platforms. For the government, the greater the flow of information, the more people involved in the relevant discussions, the more quickly the public’s unease dissipates, and the more stable society is as a consequence. The volume of traffic on the platform is related to the initial traffic, the total amount of real information on the platform, and the effort made by each subject in the “game.” For the convenience of calculation, we assume that the traffic on the social networking platform is a linear function:


(3) $$F\,\left(t\right)=f+\gamma \,\left[\lambda \,R\,\left(t\right)+\beta_{G}\,E_{G}\,\left(t\right)+\beta_{L}\,E_{L}\,\left(t\right)+\beta_{U}\,E_{U}\,\left(t\right)\right]$$

where *f* > 0 is the initial flow of information, 0 < γ≤1 is the degree of concern for major emergencies, λ > 0 is the coefficient of influence of the amount of real information on its flow, and β_*G*_ > 0, β_*L*_ > 0, and β_*U*_ > 0 are the coefficients of influence of efforts by the government, opinion leaders, and Internet users on the traffic.

Opinion leaders can benefit from traffic on the platform as well as directly from Internet users, such as by making a positive impression on them.

Assumption 5: To simplify the model, we assume that the government, opinion leaders, and Internet users have the same discount rate ρ,ρ > 0. The three entities use complete information over infinite time to maximize their own interests based on their strategic choices.

Based on assumptions 1–5, the objective functions of the government, opinion leaders, and Internet users are:


(4) $$J_{G}=\int_{0}^{\infty }e^{-\rho \,t}\,\left[\pi_{G}\,F\,\left(t\right)-C_{G}\,\left(t\right)\right]\,dt$$

(5) $$J_{L}=\int_{0}^{\infty }e^{-\rho \,t}\,\left[\omega \,E_{U}\,\left(t\right)+\pi_{L}\,F\,\left(t\right)-C_{L}\,\left(t\right)\right]\,dt$$

(6) $$J_{U}=\int_{0}^{\infty }e^{-\rho \,t}\,\left[\pi_{U}\,F\,\left(t\right)-C_{U}\,\left(t\right)\right]\,dt$$

where ω*E*_*U*_(*t*) is the income obtained by opinion leaders from Internet users, ω is the direct unit benefit to the opinion leader, π_*G*_*F*(*t*), π_*L*_*F*(*t*), and π_*U*_*F*(*t*) are revenues generated from traffic on social networking platforms by the government, opinion leaders, and Internet users, respectively, and π_*G*_, π_*L*_, and π_*U*_ are their respective marginal flow gains.

The above parameters are all constants that are independent of time. We thus omit time *t* in the subsequent derivations in this article.

## Model construction and solution

### Decentralized decision-making

In the case of decentralized decision-making, each decision-making subject of the system to correct false information makes rational decisions to maximize its own interests. To distinguish among different modes of decision-making, *N* is used to represent a decentralized decision. According to Equations 4–6, the decision-making problems for the three entities in the case of decentralized decision-making are:


(7) $$\max_{E_{G}}J_{G}^{N}=\int_{0}^{\infty }e^{-\rho \,t}\{\pi_{G}f+\pi_{G}\gamma [\lambda R+\beta_{G}E_{G}+$$

$$\beta_{L}E_{L}+\beta_{U}E_{U}]-\frac{1}{2}\mu_{G}E_{G}^{2}\}dt$$

$$\max_{E_{L}}J_{L}^{N}=\int_{0}^{\infty }e^{-\rho \,t}\{\omega E_{U}+\pi_{L}f+\pi_{L}\gamma [\lambda R+\beta_{G}E_{G}+$$

(8) $$\beta_{L}E_{L}+\beta_{U}E_{U}]-\frac{1}{2}\mu_{L}E_{L}^{2}\}dt$$

(9) $$\max_{E_{U}}J_{U}^{N}=\int_{0}^{\infty }e^{-\rho \,t}\{\pi_{U}f+\pi_{U}\gamma [\lambda R+\beta_{G}E_{G}+$$

$$\beta_{L}E_{L}+\beta_{U}E_{U}]-\frac{1}{2}\mu_{U}E_{U}^{2}\}dt$$

Theorem 1: The equilibrium results of the government, opinion leaders, and Internet users in the case of decentralized decision-making are as follows:

(1) The optimal equilibrium strategies for the government, opinion leaders, and Internet users are:


(10) $$E_{G}^{N^{*}}=\frac{\pi_{G}\,\gamma \,\left[\alpha_{G}\,\lambda +\beta_{G}\,\left(\delta +\rho \right)\right]}{\mu_{G}\,\left(\delta +\rho \right)}$$

(11) $$E_{L}^{N^{*}}=\frac{\pi_{L}\,\gamma \,\left[\alpha_{L}\,\lambda +\beta_{L}\,\left(\delta +\rho \right)\right]}{\mu_{L}\,\left(\delta +\rho \right)}$$

(12) $$E_{U}^{N^{*}}=\frac{\pi_{U}\,\gamma \,\left[\alpha_{U}\,\lambda +\beta_{U}\,\left(\delta +\rho \right)\right]}{\mu_{U}\,\left(\delta +\rho \right)}$$

(2) The optimal trajectory of the total amount of real information is:


(13) $$\left\{ \begin{array}{c} R^{N^{*}}=\left(R_{0}-R_{S}^{N}\right)\,e^{-\delta \,t}+R_{S}^{N}\\ \begin{array}{cccc} R_{S}^{N} & = & \frac{\alpha_{G}\,\pi_{G}\,\gamma \,\left[\alpha_{G}\,\lambda +\beta_{G}\,\left(\delta +\rho \right)\right]}{\delta \,\mu_{G}\,\left(\delta +\rho \right)} & \\ & & +\frac{\alpha_{L}\,\pi_{L}\,\gamma \,\left[\lambda \,\alpha_{L}+\left(\delta +\rho \right)\,\beta_{L}\right]}{\delta \,\mu_{L}\,\left(\delta +\rho \right)} & \\ & & +\frac{\alpha_{U}\,\pi_{U}\,\gamma \,\left[\lambda \,\alpha_{U}+\left(\delta +\rho \right)\,\beta_{U}\right]}{\delta \,\mu_{U}\,\left(\delta +\rho \right)} & \end{array} \end{array}\right.$$

R_*S*_*^N^* is the steady-state value of the total amount of real information under decentralized decision-making.

(3) The optimal benefits for the government, opinion leaders, and Internet users are:


(14) $$V_{G}^{N^{*}}\,\left(R\right)=\frac{\lambda \,\pi_{G}\,\gamma }{\delta +\rho }\,R_{S}^{N}+\frac{\pi_{G}\,f}{\rho }+\frac{\pi_{G}\,\gamma^{2}\,\pi_{U}\,{\left[\lambda \,\alpha_{U}+\left(\delta +\rho \right)\,\beta_{U}\right]}^{2}}{\rho \,{\left(\delta +\rho \right)}^{2}\,\mu_{U}}+$$

$$\frac{{\left[\lambda \,\pi_{G}\,\gamma \,\alpha_{G}+\pi_{G}\,\gamma \,\left(\delta +\rho \right)\,\beta_{G}\right]}^{2}}{2\,\rho \,{\left(\delta +\rho \right)}^{2}\,\mu_{G}}+\frac{\pi_{G}\,\gamma^{2}\,\pi_{L}\,{\left[\lambda \,\alpha_{L}+\left(\delta +\rho \right)\,\beta_{L}\right]}^{2}}{\rho \,{\left(\delta +\rho \right)}^{2}\,\mu_{L}}$$

$$V_{L}^{N^{*}}\,\left(R\right)=\frac{\lambda \,\pi_{L}\,\gamma }{\delta +\rho }\,R_{S}^{N}+\frac{\pi_{L}\,\gamma^{2}\,\pi_{U}\,{\left[\lambda \,\alpha_{U}+\left(\delta +\rho \right)\,\beta_{U}\right]}^{2}}{\rho \,{\left(\delta +\rho \right)}^{2}\,\mu_{U}}+$$

$$\frac{\pi_{L}\,\gamma^{2}\,\pi_{G}\,{\left[\lambda \,\alpha_{G}+\left(\delta +\rho \right)\,\beta_{G}\right]}^{2}}{\rho \,{\left(\delta +\rho \right)}^{2}\,\mu_{G}}+\frac{\gamma^{2}\,\pi_{L}^{2}\,{\left[\lambda \,\alpha_{L}+\left(\delta +\rho \right)\,\beta_{L}\right]}^{2}}{2\,\rho \,{\left(\delta +\rho \right)}^{2}\,\mu_{L}}+$$

(15) $$\frac{\gamma \,\pi_{U}\,\omega \,\left[\lambda \,\alpha_{U}+\left(\delta +\rho \right)\,\beta_{U}\right]}{\rho \,\left(\delta +\rho \right)\,\mu_{U}}+\frac{\pi_{L}\,f}{\rho }$$

$$V_{U}^{N^{*}}\,\left(R\right)=\frac{\lambda \,\pi_{U}\,\gamma }{\delta +\rho }\,R_{S}^{N}+\frac{\pi_{U}\,f}{\rho }+\frac{\pi_{U}^{2}\,\gamma^{2}\,{\left[\lambda \,\alpha_{U}+\left(\delta +\rho \right)\,\beta_{U}\right]}^{2}}{2\,\rho \,{\left(\delta +\rho \right)}^{2}\,\mu_{U}}+$$

(16) $$\frac{\pi_{U}\,\gamma^{2}\,\pi_{L}\,{\left[\lambda \,\alpha_{L}+\left(\delta +\rho \right)\,\beta_{L}\right]}^{2}}{\rho \,{\left(\delta +\rho \right)}^{2}\,\mu_{L}}+\frac{\pi_{U}\,\gamma^{2}\,\pi_{G}\,{\left[\lambda \,\alpha_{G}+\left(\delta +\rho \right)\,\beta_{G}\right]}^{2}}{\rho \,{\left(\delta +\rho \right)}^{2}\,\mu_{G}}$$

(4) The optimal benefit of the system to correct false information is:


(17) $$\begin{array}{ccc} V^{N^{*}}\,\left(R\right) & = & V_{G}^{N^{*}}\,\left(R\right)+V_{L}^{N^{*}}\,\left(R\right)+V_{U}^{N^{*}}\,\left(R\right)\\ & = & \frac{\lambda \,\gamma \,\left(\pi_{G}+\pi_{L}+\pi_{U}\right)}{\delta +\rho }\,R_{S}^{N}+\\ & & \frac{\gamma^{2}\,\pi_{L}\,\left[\pi_{L}+2\,\left(\pi_{U}+\pi_{G}\right)\right]\,{\left[\lambda \,\alpha_{L}+\left(\delta +\rho \right)\,\beta_{L}\right]}^{2}}{2\,\rho \,{\left(\delta +\rho \right)}^{2}\,\mu_{L}}+\\ & & \frac{\gamma^{2}\,\pi_{U}\,\left[\pi_{U}+2\,\left(\pi_{G}+\pi_{L}\right)\right]\,{\left[\lambda \,\alpha_{U}+\left(\delta +\rho \right)\,\beta_{U}\right]}^{2}}{2\,\rho \,{\left(\delta +\rho \right)}^{2}\,\mu_{U}}+\\ & & \frac{\gamma \,\pi_{U}\,\omega \,\left[\lambda \,\alpha_{U}+\left(\delta +\rho \right)\,\beta_{U}\right]}{\rho \,\left(\delta +\rho \right)\,\mu_{U}}+\\ & & \frac{\gamma^{2}\,\pi_{G}\,\left[\pi_{G}+2\,\left(\pi_{L}+\pi_{U}\right)\right]\,{\left[\lambda \,\alpha_{G}+\left(\delta +\rho \right)\,\beta_{G}\right]}^{2}}{2\,\rho \,{\left(\delta +\rho \right)}^{2}\,\mu_{G}}+\\ & & \frac{f\,\left(\pi_{G}+\pi_{L}+\pi_{U}\right)}{\rho } \end{array}$$

Verification 1: Given the relevant knowledge of optimal control theory in combination with Equations 7–9, the functions of the optimal benefits of the government, opinion leaders, and Internet users satisfy the HJB equation (Hamilton–Jacobi–Bellman equation):


(18) $$\rho \,V_{G}^{N}\,\left(R\right)=\max_{E_{G}}\left\{\begin{array}{c} \pi_{G}\,f+\pi_{G}\,\gamma \,\left[\lambda \,R+\beta_{G}\,E_{G}+\beta_{L}\,E_{L}+\beta_{U}\,E_{U}\right]\\ -\frac{1}{2}\,\mu_{G}\,E_{G}^{2}+V_{G}^{N^{\prime }}\,\left[\alpha_{G}\,E_{G}+\alpha_{L}\,E_{L}+\alpha_{U}\,E_{U}-\delta \,R\right] \end{array}\right\}$$

(19) $$\rho \,V_{L}^{N}\,\left(R\right)=\max_{E_{L}}\left\{\begin{array}{c} \omega \,E_{U}+\pi_{L}\,f+\pi_{L}\,\gamma \,\left[\lambda \,R+\beta_{G}\,E_{G}+\beta_{L}\,E_{L}+\beta_{U}\,E_{U}\right]\\ -\frac{1}{2}\,\mu_{L}\,E_{L}^{2}+V_{L}^{N^{\prime }}\,\left[\alpha_{G}\,E_{G}+\alpha_{L}\,E_{L}+\alpha_{U}\,E_{U}-\delta \,R\right] \end{array}\right\}$$

(20) $$\rho \,V_{U}^{N}\,\left(R\right)=\max_{E_{U}}\left\{\begin{array}{c} \pi_{U}\,f+\pi_{U}\,\gamma \,\left[\lambda \,R+\beta_{G}\,E_{G}+\beta_{L}\,E_{L}+\beta_{U}\,E_{U}\right]\\ -\frac{1}{2}\,\mu_{U}\,E_{U}^{2}+V_{U}^{N^{\prime }}\,\left[\alpha_{G}\,E_{G}+\alpha_{L}\,E_{L}+\alpha_{U}\,E_{U}-\delta \,R\right] \end{array}\right\}$$

The maximum first-order condition can be obtained by solving for E_*G*_ on the right side of Equation 18, E_*L*_ on the right side of Equation 19, and E_*U*_ on the right side of Equation 20:


(21) $$E_{G}^{N}=\frac{\pi_{G}\,\gamma \,\beta_{G}+\alpha_{G}\,V_{G}^{N^{\prime }}}{\mu_{G}}$$

(22) $$E_{L}^{N}=\frac{\pi_{L}\,\gamma \,\beta_{L}+\alpha_{L}\,V_{L}^{N^{\prime }}}{\mu_{L}}$$

(23) $$E_{U}^{N}=\frac{\pi_{U}\,\gamma \,\beta_{U}+\alpha_{U}\,V_{U}^{N^{\prime }}}{\mu_{U}}$$

The obtained Equations 21–23 can be used in Equations 18–20 to obtain the following:


(24) $$\rho \,V_{G}^{N}\,\left(R\right)$$

$$=\left(\lambda \,\pi_{G}\,\gamma -\delta \,V_{G}^{N^{\prime }}\right)\,R+\pi_{G}\,f-\frac{{\left(\alpha_{G}\,V_{G}^{N^{\prime }}+\beta_{G}\,\pi_{G}\,\gamma \right)}^{2}}{2\,\mu_{G}}$$

$$+V_{G}^{N^{\prime }}\,\left[\begin{array}{c} \frac{\alpha_{G}\,\left(\alpha_{G}\,V_{G}^{N^{\prime }}+\beta_{G}\,\pi_{G}\,\gamma \right)}{\mu_{G}}+\frac{\alpha_{L}\,\left(\alpha_{L}\,V_{L}^{N^{\prime }}+\beta_{L}\,\pi_{L}\,\gamma \right)}{\mu_{L}}\\ +\frac{\alpha_{U}\,\left(\alpha_{U}\,V_{U}^{N^{\prime }}+\beta_{U}\,\pi_{U}\,\gamma \right)}{\mu_{U}} \end{array}\right]$$

$$+\pi_{G}\,\gamma \,\left[\begin{array}{c} \frac{\beta_{G}\,\left(\alpha_{G}\,V_{G}^{N^{\prime }}+\beta_{G}\,\pi_{G}\,\gamma \right)}{\mu_{G}}+\frac{\beta_{L}\,\left(\alpha_{L}\,V_{L}^{N^{\prime }}+\beta_{L}\,\pi_{L}\,\gamma \right)}{\mu_{L}}\\ +\frac{\beta_{U}\,\left(\alpha_{U}\,V_{U}^{N^{\prime }}+\beta_{U}\,\pi_{U}\,\gamma \right)}{\mu_{U}} \end{array}\right]$$

(25) $$\begin{array}{c} \rho V_{L}^{N}\left(R\right)=\left({\lambda \pi }_{L}\gamma -\delta V_{L}^{N}{}^{\prime }\right)R-\frac{{\left(\alpha_{L}V_{L}^{N}{}^{\prime }+\beta_{L}\pi_{L}\gamma \right)}^{2}}{2\mu_{L}}+\frac{\omega \left(\alpha_{U}V_{U}^{N}{}^{\prime }+\beta_{U}\pi_{U}\gamma \right)}{\mu_{U}}+\pi_{L}f\\ +V_{L}^{N}{}^{\prime }\left[\frac{\alpha_{G}\left(\alpha_{G}V_{G}^{N}{}^{\prime }+\beta_{G}\pi_{G}\gamma \right)}{\mu_{G}}+\frac{\alpha_{L}\left(\alpha_{L}V_{L}^{N}{}^{\prime }+\beta_{L}\pi_{L}\gamma \right)}{\mu_{L}}+\frac{\alpha_{U}\left(\alpha_{U}V_{U}^{N}{}^{\prime }+\beta_{U}\pi_{U}\gamma \right)}{\mu_{U}}\right]\\ +\pi_{L}\gamma \left[\frac{\beta_{G}\left(\alpha_{G}V_{G}^{N}{}^{\prime }+\beta_{G}\pi_{G}\gamma \right)}{\mu_{G}}+\frac{\beta_{L}\left(\alpha_{L}V_{L}^{N}{}^{\prime }+\beta_{L}\pi_{L}\gamma \right)}{\mu_{L}}+\frac{\beta_{U}\left(\alpha_{U}V_{U}^{N}{}^{\prime }+\beta_{U}\pi_{U}\gamma \right)}{\mu_{U}}\right] \end{array}$$

(26) $$\begin{array}{c} \rho V_{U}^{N}\left(R\right)=\left({\lambda \pi }_{U}\gamma -\delta V_{U}^{N}{}^{\prime }\right)R+\pi_{U}f-\frac{{\left(\alpha_{U}V_{U}^{N}{}^{\prime }+\beta_{U}\pi_{U}\gamma \right)}^{2}}{2\mu_{U}}\\ +V_{U}^{N}{}^{\prime }\left[\begin{array}{l} \frac{\alpha_{G}\left(\alpha_{G}V_{G}^{N}{}^{\prime }+\beta_{G}\pi_{G}\gamma \right)}{\mu_{G}}+\frac{\alpha_{L}\left(\alpha_{L}V_{L}^{N}{}^{\prime }+\beta_{L}\pi_{L}\gamma \right)}{\mu_{L}}\\ +\frac{\alpha_{U}\left(\alpha_{U}V_{U}^{N}{}^{\prime }+\beta_{U}\pi_{U}\gamma \right)}{\mu_{U}} \end{array}\right]\\ +\pi_{U}\gamma \left[\begin{array}{l} \frac{\beta_{G}\left(\alpha_{G}V_{G}^{N}{}^{\prime }+\beta_{G}\pi_{G}\gamma \right)}{\mu_{G}}+\frac{\beta_{L}\left(\alpha_{L}V_{L}^{N}{}^{\prime }+\beta_{L}\pi_{L}\gamma \right)}{\mu_{L}}\\ +\frac{\beta_{U}\left(\alpha_{U}V_{U}^{N}{}^{\prime }+\beta_{U}\pi_{U}\gamma \right)}{\mu_{U}} \end{array}\right] \end{array}$$

By observing the structural characteristics of Equations 24–26, the analytical formulae for $V_{G}^{N}\,\left(R\right)$ and $V_{L}^{N}\,\left(R\right)$ on R can be set as follows:


(27) $$V_{G}^{N}\,\left(R\right)=a_{1}\,R+a_{2}$$

(28) $$V_{L}^{N}\,\left(R\right)=b_{1}\,R+b_{2}$$

(29) $$V_{U}^{N}\,\left(R\right)=c_{1}\,R+c_{2}$$

where a_1_, a_2_, b_1_, b_2_, c_1_, and c_2_ are constants, Further,


(30) $$V_{G}^{N^{\prime }}\,\left(R\right)=a_{1}$$

(31) $$V_{L}^{N^{\prime }}\,\left(R\right)=b_{1}$$

(32) $$V_{U}^{N^{\prime }}\,\left(R\right)=c_{1}$$

We then use the method of undetermined coefficients to import Equations 27–32 into Equations 24–26 to obtain the values of a_1_, a_2_, b_1_, b_2_, c_1_, and c_2_:


(33) $$a_{1}=\frac{\lambda \,\pi_{G}\,\gamma }{\delta +\rho }$$

$$a_{2}=\frac{\pi_{G}\,f}{\rho }+\frac{\pi_{G}\,\gamma^{2}\,\pi_{U}\,{\left[\lambda \,\alpha_{U}+\left(\delta +\rho \right)\,\beta_{U}\right]}^{2}}{\rho \,{\left(\delta +\rho \right)}^{2}\,\mu_{U}}+$$

(34) $$\frac{{\left[\lambda \,\pi_{G}\,\gamma \,\alpha_{G}+\pi_{G}\,\gamma \,\left(\delta +\rho \right)\,\beta_{G}\right]}^{2}}{2\,\rho \,{\left(\delta +\rho \right)}^{2}\,\mu_{G}}+\frac{\pi_{G}\,\gamma^{2}\,\pi_{L}\,{\left[\lambda \,\alpha_{L}+\left(\delta +\rho \right)\,\beta_{L}\right]}^{2}}{\rho \,{\left(\delta +\rho \right)}^{2}\,\mu_{L}}$$

(35) $$b_{1}=\frac{\lambda \,\pi_{L}\,\gamma }{\delta +\rho }$$

$$b_{2}=\frac{\pi_{L}\,f}{\rho }+\frac{\pi_{L}\,\gamma^{2}\,\pi_{U}\,{\left[\lambda \,\alpha_{U}+\left(\delta +\rho \right)\,\beta_{U}\right]}^{2}}{\rho \,{\left(\delta +\rho \right)}^{2}\,\mu_{U}}+$$

$$\frac{\pi_{L}\,\gamma^{2}\,\pi_{G}\,{\left[\lambda \,\alpha_{G}+\left(\delta +\rho \right)\,\beta_{G}\right]}^{2}}{\rho \,{\left(\delta +\rho \right)}^{2}\,\mu_{G}}+\frac{\gamma^{2}\,\pi_{L}^{2}\,{\left[\lambda \,\alpha_{L}+\left(\delta +\rho \right)\,\beta_{L}\right]}^{2}}{2\,\rho \,{\left(\delta +\rho \right)}^{2}\,\mu_{L}}+$$

(36) $$\frac{\gamma \,\pi_{U}\,\omega \,\left[\lambda \,\alpha_{U}+\left(\delta +\rho \right)\,\beta_{U}\right]}{\rho \,\left(\delta +\rho \right)\,\mu_{U}}$$

(37) $$c_{1}=\frac{\lambda \,\pi_{U}\,\gamma }{\delta +\rho }$$

$$c_{2}=\frac{\pi_{U}\,f}{\rho }+\frac{{\left[\lambda \,\pi_{U}\,\gamma \,\alpha_{U}+\pi_{U}\,\gamma \,\left(\delta +\rho \right)\,\beta_{U}\right]}^{2}}{2\,\rho \,{\left(\delta +\rho \right)}^{2}\,\mu_{U}}+$$

(38) $$\frac{\pi_{U}\,\gamma^{2}\,\pi_{L}\,{\left[\lambda \,\alpha_{L}+\left(\delta +\rho \right)\,\beta_{L}\right]}^{2}}{\rho \,{\left(\delta +\rho \right)}^{2}\,\mu_{L}}+\frac{\pi_{U}\,\gamma^{2}\,\pi_{G}\,{\left[\lambda \,\alpha_{G}+\left(\delta +\rho \right)\,\beta_{G}\right]}^{2}}{\rho \,{\left(\delta +\rho \right)}^{2}\,\mu_{G}}$$

By substituting the values of a_1_, b_1_, and c_1_ into Equations 21–23, the optimal equilibrium strategies of the government, opinion leaders, and Internet users can be obtained from Equations 10–12. The optimal equilibrium strategy for the three entities can then be used to obtain the optimal trajectory and steady-state value of the total amount of real information (Equation 13). By using the values of a_1_, a_2_, b_1_, b_2_, c_1_, and c_2_ in Equations 27–29, we can also obtain the optimal benefits for the government, opinion leaders, and Internet users in Equations 14–16, as well as the optimal benefits of the system to correct false information (Equation 17). Theorem 1 is thus proven.

#### Deduction 1

Theorem 1 shows that the government, opinion leaders, and Internet users make decentralized decisions. The optimal equilibrium strategy of each (i.e., their own efforts) is not affected by the other actors. An entity’s degree of effort is related only to its own parameters. The optimal trajectory of the total amount of real information is determined by these efforts. The steady-state value of the total amount of real information is positively correlated with the optimal benefits to the three entities and the entire system from correcting false information. This means that the greater the degree of effort, the greater the steady-state value of the total amount of real information, and the greater the benefits obtained by the entity itself and the entire system. The specific relationships of impact are shown in Table 1.

**TABLE 1:** The influence of the parameters on the optimal equilibrium strategies for the government, opinion leaders, and Internet users in decentralized decision-making.

	γ	π_*G*_	π_*L*_	π_*U*_	λ	α_*G*_	α_*L*_	α_*U*_	β_*G*_	β_*L*_	β_*U*_	μ_*G*_	μ_*L*_	μ_*U*_
E_*G*_^N^	+1	+1	0	0	+1	+1	0	0	+1	0	0	−1	0	0
E_*L*_^N^	+1	0	+1	0	+1	0	+1	0	0	+1	0	0	−1	0
E_*U*_^N^	+1	0	0	+1	+1	0	0	+1	0	0	+1	0	0	−1

+1 for positive impact, −1 for negative impact, and 0 for no impact.

### Centralized decision-making

Unlike in the case of decentralized decision-making, the government, opinion leaders, and Internet users jointly determine their optimal matching efforts, with the goal of maximizing the benefits to the entire system, to correct false information when making strategic choices according to centralized decision-making. To distinguish among the different modes of decision-making, C is used to represent a decentralized decision. In the case of centralized decision-making, the decision problem for the three entities can be then represented as:


(39) $$\max_{E_{G},E_{L},E_{U}}J_{S}^{C}=\int_{0}^{\infty }e^{-\rho \,t}\,\left\{\begin{array}{c} \omega \,E_{U}-\frac{1}{2}\,\mu_{G}\,E_{G}^{2}-\frac{1}{2}\,\mu_{L}\,E_{L}^{2}-\frac{1}{2}\,\mu_{U}\,E_{U}^{2}\\ +\left(\pi_{G}+\pi_{L}+\pi_{U}\right)[f+\gamma (\lambda R+\\ \beta_{G}E_{G}+\beta_{L}E_{L}+\beta_{U}E_{U})] \end{array}\right\}\,dt$$

Theorem 2: In the case of centralized decision-making, the equilibrium results of the government, opinion leaders, and Internet users are as follows:

(1) The optimal equilibrium strategies for the government, opinion leaders, and Internet users are:


(40) $$E_{G}^{C^{*}}=\frac{\gamma \,\left[\beta_{G}\,\left(\delta +\rho \right)+\alpha_{G}\,\lambda \right]\,\left(\pi_{G}+\pi_{L}+\pi_{U}\right)}{\mu_{G}\,\left(\delta +\rho \right)}$$

(41) $$E_{L}^{C^{*}}=\frac{\gamma \,\left[\beta_{L}\,\left(\delta +\rho \right)+\alpha_{L}\,\lambda \right]\,\left(\pi_{G}+\pi_{L}+\pi_{U}\right)}{\mu_{L}\,\left(\delta +\rho \right)}$$

(42) $$E_{U}^{C^{*}}=\frac{\omega \,\left(\delta +\rho \right)+\gamma \,\left[\beta_{U}\,\left(\delta +\rho \right)+\alpha_{U}\,\lambda \right]\,\left(\pi_{G}+\pi_{L}+\pi_{U}\right)}{\mu_{U}\,\left(\delta +\rho \right)}$$

(2) The optimal trajectory of the total amount of real information is:


(43) $$\left\{ \begin{array}{c} R^{C^{*}}=\left(R_{0}-R_{S}^{C}\right)\,e^{-\delta \,t}+R_{S}^{C}\\ \begin{array}{ccc} R_{S}^{C} & = & \frac{\alpha_{G}\,\gamma \,\left[\beta_{G}\,\left(\delta +\rho \right)+\alpha_{G}\,\lambda \right]\,\left(\pi_{G}+\pi_{L}+\pi_{U}\right)}{\delta \,\mu_{G}\,\left(\delta +\rho \right)}\\ & & +\frac{\alpha_{L}\,\gamma \,\left[\beta_{L}\,\left(\delta +\rho \right)+\alpha_{L}\,\lambda \right]\,\left(\pi_{G}+\pi_{L}+\pi_{U}\right)}{\delta \,\mu_{L}\,\left(\delta +\rho \right)}\\ & & +\frac{\alpha_{U}\,\omega \,\left(\delta +\rho \right)+\alpha_{U}\,\gamma \,\left[\beta_{U}\,\left(\delta +\rho \right)+\alpha_{U}\,\lambda \right]\,\left(\pi_{G}+\pi_{L}+\pi_{U}\right)}{\delta \,\mu_{U}\,\left(\delta +\rho \right)} \end{array} \end{array}\right.$$

R_*S*_*^C^* is the steady-state value of the total amount of real information under centralized decision-making.

(3) The optimal benefit for the system to correct false information is:


(44) $$V^{C^{*}}\,\left(R\right)=\frac{\lambda \,\gamma \,\left(\pi_{G}+\pi_{L}+\pi_{U}\right)}{\delta +\rho }\,R_{S}^{C}+$$

$$\frac{\gamma^{2}\,{\left(\pi_{G}+\pi_{L}+\pi_{U}\right)}^{2}\,{\left[\lambda \,\alpha_{G}+\left(\delta +\rho \right)\,\beta_{G}\right]}^{2}}{2\,\rho \,{\left(\delta +\rho \right)}^{2}\,\mu_{G}}+$$

$$\frac{\gamma^{2}\,{\left(\pi_{G}+\pi_{L}+\pi_{U}\right)}^{2}\,{\left[\lambda \,\alpha_{L}+\left(\delta +\rho \right)\,\beta_{L}\right]}^{2}}{2\,\rho \,{\left(\delta +\rho \right)}^{2}\,\mu_{L}}+$$

$$\frac{f\,\left(\pi_{G}+\pi_{L}+\pi_{U}\right)}{\rho }+\frac{\gamma^{2}\,{\left(\pi_{G}+\pi_{L}+\pi_{U}\right)}^{2}\,\left({\left[\lambda \,\alpha_{U}+\left(\delta +\rho \right)\,\beta_{U}\right]}^{2}\right)}{2\,\rho \,{\left(\delta +\rho \right)}^{2}\,\mu_{U}}$$

$$+\frac{\omega^{2}}{2\,\rho \,\mu_{U}}+\frac{\left(\pi_{G}+\pi_{L}+\pi_{U}\right)\,\gamma \,\lambda \,\omega \,\alpha_{U}}{\rho \,\left(\delta +\rho \right)\,\mu_{U}}+\frac{\gamma \,\omega \,\beta_{U}\,\left(\pi_{G}+\pi_{L}+\pi_{U}\right)}{\rho \,\mu_{U}}$$

Verification 2: Based on the knowledge of optimal control theory in combination with Equation 39, we see that the optimal utility function of the system to correct false information satisfies the HJB equation, namely,


(45) $$\rho \,V_{S}^{C}\,\left(R\right)=$$

$$\max_{E_{G},E_{L},E_{U}}\left\{\begin{array}{c} \omega \,E_{U}-\frac{1}{2}\,\mu_{G}\,E_{G}^{2}-\frac{1}{2}\,\mu_{L}\,E_{L}^{2}-\frac{1}{2}\,\mu_{U}\,E_{U}^{2}\\ +\left(\pi_{G}+\pi_{L}+\pi_{U}\right)\,\left[f+\gamma \,\left(\lambda \,R+\beta_{G}\,E_{G}+\beta_{L}\,E_{L}+\beta_{U}\,E_{U}\right)\right]\\ +V_{S}^{C^{\prime }}\,\left[\alpha_{G}\,E_{G}+\alpha_{L}\,E_{L}+\alpha_{U}\,E_{U}-\delta \,R\right] \end{array}\right\}$$

The maximum first-order conditions are used to solve for E_*G*_, E_*L*_, and E_*U*_ on the right-hand side of Equation 45:


(46) $$E_{G}^{C}=\frac{\gamma \,\beta_{G}\,\left(\pi_{G}+\pi_{L}+\pi_{U}\right)+\alpha_{G}\,V_{S}^{C^{\prime }}}{\mu_{G}}$$

(47) $$E_{L}^{C}=\frac{\gamma \,\beta_{L}\,\left(\pi_{G}+\pi_{L}+\pi_{U}\right)+\alpha_{L}\,V_{S}^{C^{\prime }}}{\mu_{L}}$$

(48) $$E_{U}^{C}=\frac{\omega +\gamma \,\beta_{U}\,\left(\pi_{G}+\pi_{L}+\pi_{U}\right)+\alpha_{U}\,V_{S}^{C^{\prime }}}{\mu_{U}}$$

By using Equations 46–48 into Equation 45, we get:


(49) $$\begin{array}{c} \text{ρ}V_{S}^{C}(R)=[\text{λγ}({\text{π}}_{G}+{\text{π}}_{L}+{\text{π}}_{U})-\text{δ}V_{S}^{C^{\prime }}]R\\ +\frac{\text{ω}[\text{ω}+{\text{α}}_{U}V_{S}^{C^{\prime }}+{\text{β}}_{U}\text{γ}({\text{π}}_{G}+{\text{π}}_{L}+{\text{π}}_{U})]}{{\text{μ}}_{U}}\\ +V_{S}^{C^{\prime }}\left\{\begin{array}{c} \frac{{\text{α}}_{G}[{\text{α}}_{G}V_{S}^{C^{\prime }}+{\text{β}}_{U}\text{γ}({\text{π}}_{G}+{\text{π}}_{L}+{\text{π}}_{U})]}{{\text{μ}}_{G}}\\ +\frac{{\text{α}}_{L}[{\text{α}}_{L}V_{S}^{C^{\prime }}+{\text{β}}_{L}\text{γ}({\text{π}}_{G}+{\text{π}}_{L}+{\text{π}}_{U})]}{{\text{μ}}_{L}}\\ +\frac{{\text{α}}_{U}[\text{ω}+{\text{α}}_{U}V_{S}^{C^{\prime }}+{\text{β}}_{U}\text{γ}({\text{π}}_{G}+{\text{π}}_{L}+{\text{π}}_{U})]}{{\text{μ}}_{U}} \end{array}\right\}\\ +({\text{π}}_{G}+{\text{π}}_{L}+{\text{π}}_{U})\\ \left\{f+\text{γ}\left[\begin{array}{c} \frac{{\text{β}}_{G}[{\text{α}}_{G}V_{S}^{C^{\prime }}+{\text{β}}_{G}\text{γ}({\text{π}}_{G}+{\text{π}}_{L}+{\text{π}}_{U})]}{{\text{μ}}_{G}}\\ +\frac{{\text{β}}_{L}[{\text{α}}_{L}V_{S}^{C^{\prime }}+{\text{β}}_{L}\text{γ}({\text{π}}_{G}+{\text{π}}_{L}+{\text{π}}_{U})]}{{\text{μ}}_{L}}\\ +\frac{{\text{β}}_{U}[\text{ω}+{\text{α}}_{U}V_{S}^{C^{\prime }}+{\text{β}}_{U}\text{γ}({\text{π}}_{G}+{\text{π}}_{L}+{\text{π}}_{U})]}{{\text{μ}}_{U}} \end{array}\right]\right\}\\ -\frac{{\left[{\text{α}}_{G}V_{S}^{C^{\prime }}+{\text{β}}_{G}\text{γ}({\text{π}}_{G}+{\text{π}}_{L}+{\text{π}}_{U})\right]}^{2}}{2{\text{μ}}_{G}}\\ -\frac{{\left[{\text{α}}_{L}V_{S}^{C^{\prime }}+{\text{β}}_{L}\text{γ}({\text{π}}_{G}+{\text{π}}_{L}+{\text{π}}_{U})\right]}^{2}}{2{\text{μ}}_{L}}\\ -\frac{{\left[\text{ω}+{\text{α}}_{U}V_{S}^{C^{\prime }}+{\text{β}}_{U}\text{γ}({\text{π}}_{G}+{\text{π}}_{L}+{\text{π}}_{U})\right]}^{2}}{2{\text{μ}}_{U}} \end{array}$$

By observing the structural characteristics of Equation 49, the analytical formula for R can be set as:


(50) $$V_{S}^{C}\,\left(R\right)=d_{1}\,R+d_{2}$$

where d_1_ and d_2_ are constants. Further,


(51) $$V_{S}^{C^{\prime }}\,\left(R\right)=d_{1}$$

By using the method of undetermined coefficients, Equations 50, 51 are imported into Equation 49 to obtain the values of d_1_ and d_2_:


(52) $$d_{1}=\frac{\lambda \,\gamma \,\left(\pi_{G}+\pi_{L}+\pi_{U}\right)}{\delta +\rho }$$

$$d_{2}=\frac{\gamma^{2}\,{\left(\pi_{G}+\pi_{L}+\pi_{U}\right)}^{2}\,{\left[\lambda \,\alpha_{G}+\left(\delta +\rho \right)\,\beta_{G}\right]}^{2}}{2\,\rho \,{\left(\delta +\rho \right)}^{2}\,\mu_{G}}+$$

$$\frac{\gamma^{2}\,{\left(\pi_{G}+\pi_{L}+\pi_{U}\right)}^{2}\,{\left[\lambda \,\alpha_{L}+\left(\delta +\rho \right)\,\beta_{L}\right]}^{2}}{2\,\rho \,{\left(\delta +\rho \right)}^{2}\,\mu_{L}}+$$

$$\frac{\gamma^{2}\,{\left(\pi_{G}+\pi_{L}+\pi_{U}\right)}^{2}\,\left({\left[\lambda \,\alpha_{U}+\left(\delta +\rho \right)\,\beta_{U}\right]}^{2}\right)}{2\,\rho \,{\left(\delta +\rho \right)}^{2}\,\mu_{U}}+$$

$$\frac{\left(\pi_{G}+\pi_{L}+\pi_{U}\right)\,\gamma \,\lambda \,\omega \,\alpha_{U}}{\rho \,\left(\delta +\rho \right)\,\mu_{U}}+\frac{\gamma \,\omega \,\beta_{U}\,\left(\pi_{G}+\pi_{L}+\pi_{U}\right)}{\rho \,\mu_{U}}+$$

(53) $$\frac{f\,\left(\pi_{G}+\pi_{L}+\pi_{U}\right)}{\rho }+\frac{\omega^{2}}{2\,\rho \,\mu_{U}}$$

By substituting the value of d_1_ into Equations 46–48, the optimal equilibrium strategies of the government, opinion leaders, and Internet users can be obtained from Equations 40–42. The optimal equilibrium strategies of the three entities can be used to obtain the optimal trajectory and steady-state value of the total amount of real information (Equation 43). We can then obtain the optimal benefits of the system to correct false information (Equation 44) by using the values of d_1_ and d_2_ in Equation 50. Theorem 2 is thus proven.

#### Deduction 2

Theorem 2 shows that the optimal equilibrium strategies (i.e., their own efforts) of the government, opinion leaders, and Internet users are also affected by other actors in the case of centralized decision-making. The degree of effort by an entity is not only related to its own parameters but also to the marginal flow of the income of the other actors. The optimal trajectory of the total amount of real information is determined by their efforts, and its steady-state value is positively correlated with the optimal benefit of the entire system to correct false information. This means that the greater the effort invested is, the greater the steady-state value of the total amount of real information, and the greater the benefits of the system. The specific relationships of impact are shown in Table 2.

**TABLE 2:** The effects of the parameters on the optimal equilibrium strategies of the government, opinion leaders, and Internet users in centralized decision-making.

	γ	π_*G*_	π_*L*_	π_*U*_	λ	α_*G*_	α_*L*_	α_*U*_	β_*G*_	β_*L*_	β_*U*_	μ_*G*_	μ_*L*_	μ_*U*_	ω
E_*G*_^N^	+1	+1	+1	+1	+1	+1	0	0	+1	0	0	−1	0	0	0
E_*L*_^N^	+1	+1	+1	+1	+1	0	+1	0	0	+1	0	0	−1	0	0
E_*U*_^N^	+1	+1	+1	+1	+1	0	0	+1	0	0	+1	0	0	−1	+1

+1 for positive impact, −1 for negative impact, and 0 for no impact.

### Cost-subsidized decision-making

The government can provide subsidies and incentives to opinion leaders and Internet users, respectively, to help correct false information online in the aftermath of major emergencies to improve their willingness to participate. We represent such incentives provided by the government by the corresponding cost-related subsidies. This scenario of subsidized decision-making constitutes a Stackelberg master–slave game, with the government as the leader, and the opinion leaders and Internet users as followers. The process of subsidized decision-making is divided into two stages. First, the government provides certain cost subsidies ε_*L*_ and ε_*U*_ to opinion leaders and Internet users, respectively. Second, the opinion leaders and Internet users choose the extent of effort to invest in spreading the correct information in light of the government’s subsidies. To distinguish among different modes of decision, S is used to represent a decision based on the given subsidy. According to Equations 4–6, the decision-making problems for the three entities in the case of cost-subsidized decision-making are:


(54) $$\max_{E_{G},\varepsilon_{L},\varepsilon_{U}}J_{G}^{S}=\int_{0}^{\infty }e^{-\rho \,t}\,\left\{\begin{array}{c} \pi_{G}\,f+\pi_{G}\,\gamma \,\left[\lambda \,R+\beta_{G}\,E_{G}+\beta_{L}\,E_{L}+\beta_{U}\,E_{U}\right]\\ -\frac{1}{2}\,\mu_{G}\,E_{G}^{2}-\frac{1}{2}\,\varepsilon_{L}\,\mu_{L}\,E_{L}^{2}-\frac{1}{2}\,\varepsilon_{U}\,\mu_{U}\,E_{U}^{2} \end{array}\right\}\,dt$$

(55) $$\max_{E_{L}}J_{L}^{S}=\int_{0}^{\infty }e^{-\rho \,t}\,\left\{\begin{array}{c} \omega \,E_{U}+\pi_{L}\,f+\pi_{L}\,\gamma \,\left[\lambda \,R+\beta_{G}\,E_{G}+\beta_{L}\,E_{L}+\beta_{U}\,E_{U}\right]\\ -\frac{1}{2}\,\left(1-\varepsilon_{L}\right)\,\mu_{L}\,E_{L}^{2} \end{array}\right\}\,dt$$

$$\max_{E_{U}}J_{U}^{S}=\int_{0}^{\infty }e^{-\rho \,t}\{\pi_{U}f+\pi_{U}\gamma [\lambda R+\beta_{G}E_{G}+\beta_{L}E_{L}$$

(56) $$+\beta_{U}E_{U}]-\frac{1}{2}\left(1-\varepsilon_{U}\right)\mu_{U}E_{U}^{2}\}dt$$

Theorem 3: In the case of decentralized decision-making, the equilibrium results of the government, opinion leaders, and Internet users are as follows:

(1) The ratios of the government’s cost-related subsidies to opinion leaders and Internet users are:


(57) $$\varepsilon_{L}^{*}=\left\{ \begin{array}{ccc} & & \frac{2\,\pi_{G}-\pi_{L}}{2\,\pi_{G}+\pi_{L}},2\,\pi_{G}>\pi_{L}\\ 0,2\,\pi_{G}<\pi_{L} & & \end{array}\right.$$

(58) $$\varepsilon_{U}^{*}=\left\{ \begin{array}{ccc} & & \frac{2\,\pi_{G}-\pi_{U}}{2\,\pi_{G}+\pi_{U}},2\,\pi_{G}>\pi_{U}\\ 0,2\,\pi_{G}<\pi_{U} & & \end{array}\right.$$

(2) The optimal equilibrium strategies for the government, opinion leaders, and Internet users are:


(59) $$E_{G}^{S^{*}}=\frac{\pi_{G}\,\gamma \,\left[\beta_{G}\,\left(\delta +\rho \right)+\alpha_{G}\,\lambda \right]}{\mu_{G}\,\left(\delta +\rho \right)}$$

(60) $$\begin{array}{ccc} E_{L}^{S^{*}} & = & \frac{\pi_{L}\,\gamma \,\left[\beta_{L}\,\left(\delta +\rho \right)+\alpha_{L}\,\lambda \right]}{\left(\delta +\rho \right)\,\mu_{L}\,\left(1-\varepsilon_{L}\right)}\\ & = & \frac{\gamma \,\left(2\,\pi_{G}+\pi_{L}\right)\,\left[\beta_{L}\,\left(\delta +\rho \right)+\alpha_{L}\,\lambda \right]}{2\,\mu_{L}\,\left(\delta +\rho \right)} \end{array}$$

(61) $$\begin{array}{ccc} E_{U}^{S^{*}} & = & \frac{\pi_{U}\,\gamma \,\left[\beta_{U}\,\left(\delta +\rho \right)+\alpha_{U}\,\lambda \right]}{\mu_{U}\,\left(1-\varepsilon_{U}\right)\,\left(\delta +\rho \right)}\\ & = & \frac{\gamma \,\left(2\,\pi_{G}+\pi_{U}\right)\,\left[\beta_{U}\,\left(\delta +\rho \right)+\alpha_{U}\,\lambda \right]}{2\,\mu_{U}\,\left(\delta +\rho \right)} \end{array}$$

(3) The optimal trajectory of the total amount of real information is:


(62) $$\begin{array}{ccc} & & \left\{ \begin{array}{c} R^{S^{*}}=\left(R_{0}-R_{S}^{S}\right)\,e^{-\delta \,t}+R_{S}^{S}\\ \begin{array}{ccc} R_{S}^{S} & = & \frac{\alpha_{G}\,\pi_{G}\,\gamma \,\left[\beta_{G}\,\left(\delta +\rho \right)+\alpha_{G}\,\lambda \right]}{\delta \,\mu_{G}\,\left(\delta +\rho \right)}\\ & & +\frac{\alpha_{L}\,\pi_{L}\,\gamma \,\left[\beta_{L}\,\left(\delta +\rho \right)+\alpha_{L}\,\lambda \right]}{\delta \,\left(\delta +\rho \right)\,\mu_{L}\,\left(1-\varepsilon_{L}\right)}\\ & & +\frac{\alpha_{U}\,\pi_{U}\,\gamma \,\left[\beta_{U}\,\left(\delta +\rho \right)+\alpha_{U}\,\lambda \right]}{\delta \,\mu_{U}\,\left(1-\varepsilon_{U}\right)\,\left(\delta +\rho \right)}\\ & = & \frac{\alpha_{G}\,\pi_{G}\,\gamma \,\left[\beta_{G}\,\left(\delta +\rho \right)+\alpha_{G}\,\lambda \right]}{\delta \,\mu_{G}\,\left(\delta +\rho \right)}\\ & & +\frac{\alpha_{L}\,\gamma \,\left(2\,\pi_{G}+\pi_{L}\right)\,\left[\beta_{L}\,\left(\delta +\rho \right)+\alpha_{L}\,\lambda \right]}{2\,\delta \,\mu_{L}\,\left(\delta +\rho \right)}\\ & & +\frac{\alpha_{U}\,\gamma \,\left(2\,\pi_{G}+\pi_{U}\right)\,\left[\beta_{U}\,\left(\delta +\rho \right)+\alpha_{U}\,\lambda \right]}{2\,\delta \,\mu_{U}\,\left(\delta +\rho \right)} \end{array} \end{array}\right. \end{array}$$

R_*S*_*^S^* is the value of the total amount of real information under cost-subsidized decision-making.

(4) The optimal benefits for the government, opinion leaders, and Internet users are:

$$V_{G}^{S^{*}}\,\left(R\right)=\frac{\lambda \,\pi_{G}\,\gamma }{\delta +\rho }\,R_{S}^{S}+\frac{\gamma^{2}\,\pi_{G}^{2}\,{\left[\lambda \,\alpha_{G}+\left(\delta +\rho \right)\,\beta_{G}\right]}^{2}}{2\,\rho \,{\left(\delta +\rho \right)}^{2}\,\mu_{G}}+\frac{\pi_{G}\,f}{\rho }+$$

$$\frac{\pi_{G}\,\pi_{U}\,\gamma^{2}\,{\left[\lambda \,\alpha_{U}+\left(\delta +\rho \right)\,\beta_{U}\right]}^{2}}{\rho \,{\left(\delta +\rho \right)}^{2}\,\left(1-\varepsilon_{U}\right)\,\mu_{U}}+\frac{\pi_{G}\,\pi_{L}\,\gamma^{2}\,{\left[\lambda \,\alpha_{L}+\left(\delta +\rho \right)\,\beta_{L}\right]}^{2}}{\rho \,{\left(\delta +\rho \right)}^{2}\,\left(1-\varepsilon_{L}\right)\,\mu_{L}}$$

$$-\frac{\gamma^{2}\,\pi_{U}^{2}\,\varepsilon_{U}\,{\left[\lambda \,\alpha_{U}+\left(\delta +\rho \right)\,\beta_{U}\right]}^{2}}{2\,\rho \,{\left(\delta +\rho \right)}^{2}\,{\left(1-\varepsilon_{U}\right)}^{2}\,\mu_{U}}-\frac{\gamma^{2}\,\pi_{L}^{2}\,\varepsilon_{L}\,{\left[\lambda \,\alpha_{L}+\left(\delta +\rho \right)\,\beta_{L}\right]}^{2}}{2\,\rho \,{\left(\delta +\rho \right)}^{2}\,{\left(1-\varepsilon_{L}\right)}^{2}\,\mu_{L}}$$

$$=\frac{\lambda \,\pi_{G}\,\gamma }{\delta +\rho }\,R_{S}^{S}+\frac{\gamma^{2}\,{\left(\pi_{L}+2\,\pi_{G}\right)}^{2}\,{\left[\lambda \,\alpha_{L}+\left(\delta +\rho \right)\,\beta_{L}\right]}^{2}}{8\,\rho \,{\left(\delta +\rho \right)}^{2}\,\mu_{L}}+$$

$$\frac{\gamma^{2}\,\pi_{G}^{2}\,{\left[\lambda \,\alpha_{G}+\left(\delta +\rho \right)\,\beta_{G}\right]}^{2}}{2\,\rho \,{\left(\delta +\rho \right)}^{2}\,\mu_{G}}+\frac{f\,\pi_{G}}{\rho }+$$

(63) $$\frac{\gamma^{2}\,{\left(\pi_{U}+2\,\pi_{G}\right)}^{2}\,{\left[\lambda \,\alpha_{U}+\left(\delta +\rho \right)\,\beta_{U}\right]}^{2}}{8\,\rho \,{\left(\delta +\rho \right)}^{2}\,\mu_{U}}$$

$$V_{L}^{S^{*}}\,\left(R\right)=\frac{\lambda \,\pi_{L}\,\gamma }{\delta +\rho }\,R_{S}^{S}+\frac{\gamma \,\omega \,\pi_{U}\,\left[\lambda \,\alpha_{U}+\left(\delta +\rho \right)\,\beta_{U}\right]}{\rho \,\left(\delta +\rho \right)\,\left(1-\varepsilon_{U}\right)\,\mu_{U}}+$$

$$\frac{\gamma^{2}\,\pi_{G}\,\pi_{L}\,{\left[\lambda \,\alpha_{G}+\left(\delta +\rho \right)\,\beta_{G}\right]}^{2}}{\rho \,\mu_{G}\,{\left(\delta +\rho \right)}^{2}}+$$

$$\frac{\gamma^{2}\,\pi_{U}\,\pi_{L}\,{\left[\lambda \,\alpha_{U}+\left(\delta +\rho \right)\,\beta_{U}\right]}^{2}}{\rho \,\left(1-\varepsilon_{U}\right)\,\mu_{U}\,{\left(\delta +\rho \right)}^{2}}+$$

$$\frac{\pi_{L}\,f}{\rho }+\frac{\gamma^{2}\,\pi_{L}^{2}\,{\left[\lambda \,\alpha_{L}+\left(\delta +\rho \right)\,\beta_{L}\right]}^{2}}{2\,\rho \,{\left(\delta +\rho \right)}^{2}\,\left(1-\varepsilon_{L}\right)\,\mu_{L}}=$$

$$\frac{\lambda \,\pi_{L}\,\gamma }{\delta +\rho }\,R_{S}^{S}+\frac{\gamma^{2}\,\pi_{G}\,\pi_{L}\,{\left[\lambda \,\alpha_{G}+\left(\delta +\rho \right)\,\beta_{G}\right]}^{2}}{\rho \,{\left(\delta +\rho \right)}^{2}\,\mu_{G}}+$$

$$\frac{f\,\pi_{L}}{\rho }+\frac{\gamma^{2}\,\pi_{L}\,\left(2\,\pi_{G}+\pi_{L}\right)\,{\left[\lambda \,\alpha_{L}+\left(\delta +\rho \right)\,\beta_{L}\right]}^{2}}{4\,\rho \,{\left(\delta +\rho \right)}^{2}\,\mu_{L}}+$$

$$\frac{\omega \,\gamma \,\left(2\,\pi_{G}+\pi_{U}\right)\,\left[\lambda \,\alpha_{U}+\left(\delta +\rho \right)\,\beta_{U}\right]}{2\,\rho \,\left(\delta +\rho \right)\,\mu_{U}}+$$

(64) $$\frac{\gamma^{2}\,\pi_{L}\,\left(2\,\pi_{G}+\pi_{U}\right)\,{\left[\lambda \,\alpha_{U}+\left(\delta +\rho \right)\,\beta_{U}\right]}^{2}}{2\,\rho \,{\left(\delta +\rho \right)}^{2}\,\mu_{U}}$$

$$V_{U}^{S^{*}}\,\left(R\right)=\frac{\lambda \,\pi_{U}\,\gamma }{\delta +\rho }\,R_{S}^{S}+\frac{\gamma^{2}\,\pi_{U}^{2}\,{\left[\lambda \,\alpha_{U}+\left(\delta +\rho \right)\,\beta_{U}\right]}^{2}}{2\,\rho \,\left(1-\varepsilon_{U}\right)\,\mu_{U}\,{\left(\delta +\rho \right)}^{2}}+$$

$$\frac{\gamma^{2}\,\pi_{G}\,\pi_{U}\,{\left[\lambda \,\alpha_{G}+\left(\delta +\rho \right)\,\beta_{G}\right]}^{2}}{\mu_{G}\,\rho \,{\left(\delta +\rho \right)}^{2}}+$$

$$\frac{\gamma^{2}\,\pi_{L}\,\pi_{U}\,{\left[\lambda \,\alpha_{L}+\left(\delta +\rho \right)\,\beta_{L}\right]}^{2}}{\rho \,\left(1-\varepsilon_{L}\right)\,\mu_{L}\,{\left(\delta +\rho \right)}^{2}}+$$

$$\frac{f\,\pi_{U}}{\rho }=\frac{\lambda \,\pi_{U}\,\gamma }{\delta +\rho }\,R_{S}^{S}+$$

$$\frac{\gamma^{2}\,\pi_{G}\,\pi_{U}\,{\left[\lambda \,\alpha_{G}+\left(\delta +\rho \right)\,\beta_{G}\right]}^{2}}{\rho \,{\left(\delta +\rho \right)}^{2}\,\mu_{G}}+\frac{\pi_{U}\,f}{\rho }+$$

$$\frac{\gamma^{2}\,\pi_{U}\,\left(2\,\pi_{G}+\pi_{L}\right)\,{\left[\lambda \,\alpha_{L}+\left(\delta +\rho \right)\,\beta_{L}\right]}^{2}}{2\,\rho \,{\left(\delta +\rho \right)}^{2}\,\mu_{L}}+$$

(65) $$\frac{\gamma^{2}\,\pi_{U}\,\left(2\,\pi_{G}+\pi_{U}\right)\,{\left[\lambda \,\alpha_{U}+\left(\delta +\rho \right)\,\beta_{U}\right]}^{2}}{4\,\rho \,{\left(\delta +\rho \right)}^{2}\,\mu_{U}}$$

(5) The optimal benefit of the system to correct false information is:

$$V^{S^{*}}\,\left(R\right)=V_{G}^{S^{*}}\,\left(R\right)+V_{L}^{S^{*}}\,\left(R\right)+V_{U}^{S^{*}}$$

$$\left(R\right)=\frac{\lambda \,\gamma \,\left(\pi_{G}+\pi_{L}+\pi_{U}\right)}{\delta +\rho }\,R_{S}^{S}+\frac{\gamma^{2}\,\pi_{G}^{2}\,{\left[\lambda \,\alpha_{G}+\left(\delta +\rho \right)\,\beta_{G}\right]}^{2}}{2\,\rho \,{\left(\delta +\rho \right)}^{2}\,\mu_{G}}+$$

$$\frac{f\,\left(\pi_{G}+\pi_{L}+\pi_{U}\right)}{\rho }+\frac{\gamma^{2}\,\pi_{G}\,\left(\pi_{U}+\pi_{L}\right)\,{\left[\lambda \,\alpha_{G}+\left(\delta +\rho \right)\,\beta_{G}\right]}^{2}}{\rho \,\mu_{G}\,{\left(\delta +\rho \right)}^{2}}-$$

$$\frac{\gamma^{2}\,\pi_{U}^{2}\,\varepsilon_{U}\,{\left[\lambda \,\alpha_{U}+\left(\delta +\rho \right)\,\beta_{U}\right]}^{2}}{2\,\rho \,{\left(\delta +\rho \right)}^{2}\,{\left(1-\varepsilon_{U}\right)}^{2}\,\mu_{U}}+$$

$$\frac{\gamma^{2}\,\pi_{L}\,\left(2\,\pi_{G}+2\,\pi_{U}+\pi_{L}\right)\,{\left[\lambda \,\alpha_{L}+\left(\delta +\rho \right)\,\beta_{L}\right]}^{2}}{2\,\rho \,{\left(\delta +\rho \right)}^{2}\,\left(1-\varepsilon_{L}\right)\,\mu_{L}}-$$

$$\frac{\gamma^{2}\,\pi_{L}^{2}\,\varepsilon_{L}\,{\left[\lambda \,\alpha_{L}+\left(\delta +\rho \right)\,\beta_{L}\right]}^{2}}{2\,\rho \,{\left(\delta +\rho \right)}^{2}\,{\left(1-\varepsilon_{L}\right)}^{2}\,\mu_{L}}+\frac{\gamma \,\omega \,\pi_{U}\,\left[\lambda \,\alpha_{U}+\left(\delta +\rho \right)\,\beta_{U}\right]}{\rho \,\left(\delta +\rho \right)\,\left(1-\varepsilon_{U}\right)\,\mu_{U}}+$$

$$\frac{\gamma^{2}\,\pi_{U}\,\left(2\,\pi_{G}+2\,\pi_{L}+\pi_{U}\right)\,{\left[\lambda \,\alpha_{U}+\left(\delta +\rho \right)\,\beta_{U}\right]}^{2}}{2\,\rho \,{\left(\delta +\rho \right)}^{2}\,\left(1-\varepsilon_{U}\right)\,\mu_{U}}=$$

$$\frac{\lambda \,\gamma \,\left(\pi_{G}+\pi_{L}+\pi_{U}\right)}{\delta +\rho }\,R_{S}^{S}+$$

$$\frac{\gamma^{2}\,\pi_{G}\,\left(\pi_{G}+2\,\pi_{L}+2\,\pi_{U}\right)\,{\left[\lambda \,\alpha_{G}+\left(\delta +\rho \right)\,\beta_{G}\right]}^{2}}{2\,\rho \,{\left(\delta +\rho \right)}^{2}\,\mu_{G}}+$$

$$\frac{\gamma^{2}\,\left(2\,\pi_{G}+\pi_{L}\right)\,\left(2\,\pi_{G}+3\,\pi_{L}+4\,\pi_{U}\right)\,{\left[\lambda \,\alpha_{L}+\left(\delta +\rho \right)\,\beta_{L}\right]}^{2}}{8\,\rho \,{\left(\delta +\rho \right)}^{2}\,\mu_{L}}+$$

$$\frac{\gamma^{2}\,\left(2\,\pi_{G}+\pi_{U}\right)\,\left(2\,\pi_{G}+4\,\pi_{L}+3\,\pi_{U}\right)\,{\left[\lambda \,\alpha_{U}+\left(\delta +\rho \right)\,\beta_{U}\right]}^{2}}{8\,\rho \,{\left(\delta +\rho \right)}^{2}\,\mu_{U}}+$$

(66) $$\frac{\omega \,\gamma \,\left(2\,\pi_{G}+\pi_{U}\right)\,\left[\lambda \,\alpha_{U}+\left(\delta +\rho \right)\,\beta_{U}\right]}{2\,\rho \,\left(\delta +\rho \right)\,\mu_{U}}+\frac{f\,\left(\pi_{G}+\pi_{L}+\pi_{U}\right)}{\rho }$$

Verification 3: Based on the knowledge of optimal control theory, we used backward induction. The problem of the optimal control of opinion leaders and Internet users is first studied. The HJB equations of the optimal benefit functions of opinion leaders and Internet users are:

$$\rho \,V_{L}^{S}\,\left(R\right)=$$

(67) $$\max_{E_{L}}\left\{\begin{array}{c} \omega \,E_{U}+\pi_{L}\,f+\pi_{L}\,\gamma \,\left[\lambda \,R+\beta_{G}\,E_{G}+\beta_{L}\,E_{L}+\beta_{U}\,E_{U}\right]\\ -\frac{1}{2}\,\left(1-\varepsilon_{L}\right)\,\mu_{L}\,E_{L}^{2}+V_{L}^{S^{\prime }}\,\left[\alpha_{G}\,E_{G}+\alpha_{L}\,E_{L}+\alpha_{U}\,E_{U}-\delta \,R\right] \end{array}\right\}$$

$$\rho \,V_{U}^{S}\,\left(R\right)=$$

(68) $$\max_{E_{U}}\left\{\begin{array}{c} \pi_{U}\,f+\pi_{U}\,\gamma \,\left[\lambda \,R+\beta_{G}\,E_{G}+\beta_{L}\,E_{L}+\beta_{U}\,E_{U}\right]\\ -\frac{1}{2}\,\left(1-\varepsilon_{U}\right)\,\mu_{U}\,E_{U}^{2}+V_{U}^{S^{\prime }}\,\left[\alpha_{G}\,E_{G}+\alpha_{L}\,E_{L}+\alpha_{U}\,E_{U}-\delta \,R\right] \end{array}\right\}$$

The maximum first-order condition can be obtained by solving for E_*L*_ on the right side of Equation 67 and E_*U*_ on the right side of Equation 68:


(69) $$E_{L}^{S}=\frac{\pi_{L}\,\gamma \,\beta_{L}+\alpha_{L}\,V_{L}^{S^{\prime }}}{\mu_{L}\,\left(1-\varepsilon_{L}\right)}$$

(70) $$E_{U}^{S}=\frac{\pi_{U}\,\gamma \,\beta_{U}+\alpha_{U}\,V_{U}^{S^{\prime }}}{\mu_{U}\,\left(1-\varepsilon_{U}\right)}$$

The problem of optimal control of the government is then analyzed. The HJB equation of the government’s optimal benefit function is:


(71) $$\rho \,V_{G}^{S}\,\left(R\right)=\max_{E_{G},\varepsilon_{L},\varepsilon_{U}}\left\{\begin{array}{c} \pi_{G}\,f+\pi_{G}\,\gamma \,\left[\lambda \,R+\beta_{G}\,E_{G}+\beta_{L}\,E_{L}+\beta_{U}\,E_{U}\right]\\ -\frac{1}{2}\,\mu_{G}\,E_{G}^{2}-\frac{1}{2}\,\varepsilon_{L}\,\mu_{L}\,E_{L}^{2}-\frac{1}{2}\,\varepsilon_{U}\,\mu_{U}\,E_{U}^{2}\\ +V_{G}^{S^{\prime }}\,\left[\alpha_{G}\,E_{G}+\alpha_{L}\,E_{L}+\alpha_{U}\,E_{U}-\delta \,R\right] \end{array}\right\}$$

The maximum first-order condition can be obtained by solving for E_*G*_ on the right side of Equation 71:


(72) $$E_{G}^{S}=\frac{\pi_{G}\,\gamma \,\beta_{G}+\alpha_{G}\,V_{G}^{S^{\prime }}}{\mu_{G}}$$

Bringing Equations 69, 70 into Equation 71 yields:


(73) $$\rho \,V_{G}^{S}\,\left(R\right)=\max_{E_{G},\varepsilon_{L},\varepsilon_{U}}\left\{\begin{array}{c} \pi_{G}\,f-\frac{1}{2}\,\mu_{G}\,E_{G}^{2}-\frac{1}{2}\,\varepsilon_{L}\,\mu_{L}\,{\left[\frac{\pi_{L}\,\gamma \,\beta_{L}+\alpha_{L}\,V_{L}^{S^{\prime }}}{\mu_{L}\,\left(1-\varepsilon_{L}\right)}\right]}^{2}\\ +\pi_{G}\,\gamma \,\left[\begin{array}{c} \lambda \,R+\frac{\beta_{L}\,\left(\pi_{L}\,\gamma \,\beta_{L}+\alpha_{L}\,V_{L}^{S^{\prime }}\right)}{\mu_{L}\,\left(1-\varepsilon_{L}\right)}\\ +\frac{\beta_{U}\,\left(\pi_{U}\,\gamma \,\beta_{U}+\alpha_{U}\,V_{U}^{S^{\prime }}\right)}{\mu_{U}\,\left(1-\varepsilon_{U}\right)}+\beta_{G}\,E_{G} \end{array}\right]\\ -\frac{1}{2}\,\varepsilon_{U}\,\mu_{U}\,{\left[\frac{\pi_{U}\,\gamma \,\beta_{U}+\alpha_{U}\,V_{U}^{S^{\prime }}}{\mu_{U}\,\left(1-\varepsilon_{U}\right)}\right]}^{2}\\ +V_{G}^{S^{\prime }}\,\left[\begin{array}{c} \alpha_{G}\,E_{G}+\frac{\alpha_{L}\,\left(\pi_{L}\,\gamma \,\beta_{L}+\alpha_{L}\,V_{L}^{S^{\prime }}\right)}{\mu_{L}\,\left(1-\varepsilon_{L}\right)}\\ +\frac{\alpha_{U}\,\left(\pi_{U}\,\gamma \,\beta_{U}+\alpha_{U}\,V_{U}^{S^{\prime }}\right)}{\mu_{U}\,\left(1-\varepsilon_{U}\right)}-\delta \,R \end{array}\right] \end{array}\right\}$$

The maximum first-order conditions are obtained by solving for ε_*L*_ and ε_*U*_ on the right side of Equation 73:


(74) $$\varepsilon_{L}=\left\{ \begin{array}{cc} & \frac{2\,\left(\alpha_{L}\,V_{G}^{S^{\prime }}+\gamma \,\beta_{L}\,\pi_{G}\right)-\left(\gamma \,\beta_{L}\,\pi_{L}+\alpha_{L}\,V_{L}^{S^{\prime }}\right)}{2\,\left(\alpha_{L}\,V_{G}^{S^{\prime }}+\gamma \,\beta_{L}\,\pi_{G}\right)+\left(\gamma \,\beta_{L}\,\pi_{L}+\alpha_{L}\,V_{L}^{S^{\prime }}\right)},B_{L}>C_{L}\\ 0,B_{L}<C_{L} & \end{array}\right.$$

$B_{L}=2\,\left(\alpha_{L}\,V_{G}^{S^{\prime }}+\gamma \,\beta_{L}\,\pi_{G}\right),C_{L}=\gamma \,\beta_{L}\,\pi_{L}+\alpha_{L}\,V_{L}^{S^{\prime }}$.


(75) $$\varepsilon_{U}=\left\{ \begin{array}{ccc} & & \frac{2\,\left(\alpha_{U}\,V_{G}^{S^{\prime }}+\gamma \,\beta_{U}\,\pi_{G}\right)-\left(\gamma \,\beta_{U}\,\pi_{U}+\alpha_{U}\,V_{U}^{S^{\prime }}\right)}{2\,\left(\alpha_{U}\,V_{G}^{S^{\prime }}+\gamma \,\beta_{U}\,\pi_{G}\right)+\left(\gamma \,\beta_{U}\,\pi_{U}+\alpha_{U}\,V_{U}^{S^{\prime }}\right)},B_{U}>C_{U}\\ 0,B_{U}<C_{U} & & \end{array}\right.$$

$B_{U}=2\,\left(\alpha_{U}\,V_{G}^{S^{\prime }}+\gamma \,\beta_{U}\,\pi_{U}\right),C_{U}=\gamma \,\beta_{U}\,\pi_{U}+\alpha_{U}\,V_{U}^{S^{\prime }}$.

Equations 69, 70, and 72 are used in Equations 67, 68, and 71 to obtain the following:


(76) $$\begin{array}{ccc} \rho \,V_{L}^{S}\,\left(R\right) & = & \left(\lambda \,\pi_{L}\,\gamma -\delta \,V_{L}^{S^{\prime }}\right)\,R+\frac{\omega \,\left(\alpha_{U}\,V_{U}^{S^{\prime }}+\beta_{U}\,\pi_{U}\,\gamma \right)}{\mu_{U}\,\left(1-\varepsilon_{U}\right)}\\ & & +V_{L}^{S^{\prime }}\,\left[\begin{array}{c} \frac{\alpha_{L}\,\left(\alpha_{L}\,V_{L}^{S^{\prime }}+\beta_{L}\,\pi_{L}\,\gamma \right)}{\mu_{L}\,\left(1-\varepsilon_{L}\right)}\\ +\frac{\alpha_{U}\,\left(\alpha_{U}\,V_{U}^{S^{\prime }}+\beta_{U}\,\pi_{U}\,\gamma \right)}{\mu_{U}\,\left(1-\varepsilon_{U}\right)}\\ +\frac{\alpha_{G}\,\left(\alpha_{G}\,V_{G}^{S^{\prime }}+\beta_{G}\,\pi_{G}\,\gamma \right)}{\mu_{G}} \end{array}\right]+\pi_{L}\,f\\ & & +\pi_{L}\,\gamma \,\left[\begin{array}{c} \frac{\beta_{G}\,\left(\alpha_{G}\,V_{G}^{S^{\prime }}+\beta_{G}\,\pi_{G}\,\gamma \right)}{\mu_{G}}\\ +\frac{\beta_{L}\,\left(\alpha_{L}\,V_{L}^{S^{\prime }}+\beta_{L}\,\pi_{L}\,\gamma \right)}{\mu_{L}\,\left(1-\varepsilon_{L}\right)}\\ +\frac{\beta_{U}\,\left(\alpha_{U}\,V_{U}^{S^{\prime }}+\beta_{U}\,\pi_{U}\,\gamma \right)}{\mu_{U}\,\left(1-\varepsilon_{U}\right)} \end{array}\right]\\ & & +\frac{\varepsilon_{L}\,\mu_{L}\,{\left(\alpha_{L}\,V_{L}^{S^{\prime }}+\beta_{L}\,\pi_{L}\,\gamma \right)}^{2}}{2\,\mu_{L}^{2}\,{\left(1-\varepsilon_{L}\right)}^{2}}-\frac{\mu_{L}\,{\left(\alpha_{L}\,V_{L}^{S^{\prime }}+\beta_{L}\,\pi_{L}\,\gamma \right)}^{2}}{2\,\mu_{L}^{2}\,{\left(1-\varepsilon_{L}\right)}^{2}} \end{array}$$

$$\rho \,V_{U}^{S}\,\left(R\right)=\left(\lambda \,\pi_{U}\,\gamma -\delta \,V_{U}^{S^{\prime }}\right)\,R+\pi_{U}\,f-$$

$$\frac{\mu_{U}\,{\left(\alpha_{U}\,V_{U}^{S^{\prime }}+\beta_{U}\,\pi_{U}\,\gamma \right)}^{2}}{2\,\mu_{U}^{2}\,{\left(1-\varepsilon_{U}\right)}^{2}}+\frac{\varepsilon_{U}\,\mu_{U}\,{\left(\alpha_{U}\,V_{U}^{S^{\prime }}+\beta_{U}\,\pi_{U}\,\gamma \right)}^{2}}{2\,\mu_{U}^{2}\,{\left(1-\varepsilon_{U}\right)}^{2}}+$$

$$\pi_{U}\,\gamma \,\left[\begin{array}{c} \frac{\beta_{G}\,\left(\alpha_{G}\,V_{G}^{S^{\prime }}+\beta_{G}\,\pi_{G}\,\gamma \right)}{\mu_{G}}+\frac{\beta_{L}\,\left(\alpha_{L}\,V_{L}^{S^{\prime }}+\beta_{L}\,\pi_{L}\,\gamma \right)}{\mu_{L}\,\left(1-\varepsilon_{L}\right)}\\ +\frac{\beta_{U}\,\left(\alpha_{U}\,V_{U}^{S^{\prime }}+\beta_{U}\,\pi_{U}\,\gamma \right)}{\mu_{U}\,\left(1-\varepsilon_{U}\right)} \end{array}\right]+$$

(77) $$V_{U}^{S^{\prime }}\,\left[\begin{array}{c} \frac{\alpha_{L}\,\left(\alpha_{L}\,V_{L}^{S^{\prime }}+\beta_{L}\,\pi_{L}\,\gamma \right)}{\mu_{L}\,\left(1-\varepsilon_{L}\right)}+\frac{\alpha_{U}\,\left(\alpha_{U}\,V_{U}^{S^{\prime }}+\beta_{U}\,\pi_{U}\,\gamma \right)}{\mu_{U}\,\left(1-\varepsilon_{U}\right)}\\ +\frac{\alpha_{G}\,\left(\alpha_{G}\,V_{G}^{S^{\prime }}+\beta_{G}\,\pi_{G}\,\gamma \right)}{\mu_{G}} \end{array}\right]$$

$$\rho \,V_{G}^{S}\,\left(R\right)=\left(\lambda \,\pi_{G}\,\gamma -\delta \,V_{G}^{S^{\prime }}\right)\,R+\pi_{G}\,f-\frac{{\left(\alpha_{G}\,V_{G}^{S^{\prime }}+\beta_{G}\,\pi_{G}\,\gamma \right)}^{2}}{2\,\mu_{G}}+$$

$$V_{G}^{S^{\prime }}\,\left[\begin{array}{c} \frac{\alpha_{L}\,\left(\alpha_{L}\,V_{L}^{S^{\prime }}+\beta_{L}\,\pi_{L}\,\gamma \right)}{\mu_{L}\,\left(1-\varepsilon_{L}\right)}+\frac{\alpha_{U}\,\left(\alpha_{U}\,V_{U}^{S^{\prime }}+\beta_{U}\,\pi_{U}\,\gamma \right)}{\mu_{U}\,\left(1-\varepsilon_{U}\right)}\\ +\frac{\alpha_{G}\,\left(\alpha_{G}\,V_{G}^{S^{\prime }}+\beta_{G}\,\pi_{G}\,\gamma \right)}{\mu_{G}} \end{array}\right]+\pi_{G}\,\gamma$$

$$\left[\begin{array}{c} \frac{\beta_{G}\,\left(\alpha_{G}\,V_{G}^{S^{\prime }}+\beta_{G}\,\pi_{G}\,\gamma \right)}{\mu_{G}}+\frac{\beta_{U}\,\left(\alpha_{U}\,V_{U}^{S^{\prime }}+\beta_{U}\,\pi_{U}\,\gamma \right)}{\mu_{U}\,\left(1-\varepsilon_{U}\right)}\\ +\frac{\beta_{L}\,\left(\alpha_{L}\,V_{L}^{S^{\prime }}+\beta_{L}\,\pi_{L}\,\gamma \right)}{\mu_{L}\,\left(1-\varepsilon_{L}\right)} \end{array}\right]$$

(78) $$-\frac{\varepsilon_{L}\,{\left(\alpha_{L}\,V_{L}^{S^{\prime }}+\beta_{L}\,\pi_{L}\,\gamma \right)}^{2}}{2\,\mu_{L}\,{\left(1-\varepsilon_{L}\right)}^{2}}-\frac{\varepsilon_{U}\,{\left(\alpha_{U}\,V_{U}^{S^{\prime }}+\beta_{U}\,\pi_{U}\,\gamma \right)}^{2}}{2\,\mu_{U}\,{\left(1-\varepsilon_{U}\right)}^{2}}$$

By observing the structural characteristics of Equations 76–78, the analytical formulae for $V_{G}^{S}\,\left(R\right)$ and $V_{L}^{S}\,\left(R\right)$ on R can be given as follows:


(79) $$V_{G}^{S}\,\left(R\right)=i_{1}\,R+i_{2}$$

(80) $$V_{L}^{S}\,\left(R\right)=j_{1}\,R+j_{2}$$

(81) $$V_{U}^{S}\,\left(R\right)=k_{1}\,R+k_{2}$$

where i_1_, i_2_, j_1_, j_2_, k_1_, and k_2_ are constants. Furthermore,


(82) $$V_{G}^{S^{\prime }}\,\left(R\right)=i_{1}$$

(83) $$V_{G}^{S^{\prime }}\,\left(R\right)=i_{1}$$

(84) $$V_{U}^{S^{\prime }}\,\left(R\right)=k_{1}$$

By using the method of undetermined coefficients, Equations 79–84 are used in Equations 76–78 to obtain the values of i_1_, i_2_, j_1_, j_2_, k_1_, and k_2_:


(85) $$i_{1}=\frac{\lambda \,\pi_{G}\,\gamma }{\delta +\rho }$$

$$i_{2}=\frac{\gamma^{2}\,\pi_{G}^{2}\,{\left[\lambda \,\alpha_{G}+\left(\delta +\rho \right)\,\beta_{G}\right]}^{2}}{2\,\rho \,{\left(\delta +\rho \right)}^{2}\,\mu_{G}}+\frac{\pi_{G}\,f}{\rho }+$$

$$\frac{\pi_{G}\,\pi_{U}\,\gamma^{2}\,{\left[\lambda \,\alpha_{U}+\left(\delta +\rho \right)\,\beta_{U}\right]}^{2}}{\rho \,{\left(\delta +\rho \right)}^{2}\,\left(1-\varepsilon_{U}\right)\,\mu_{U}}+\frac{\pi_{G}\,\pi_{L}\,\gamma^{2}\,{\left[\lambda \,\alpha_{L}+\left(\delta +\rho \right)\,\beta_{L}\right]}^{2}}{\rho \,{\left(\delta +\rho \right)}^{2}\,\left(1-\varepsilon_{L}\right)\,\mu_{L}}$$

(86) $$-\frac{\gamma^{2}\,\pi_{U}^{2}\,\varepsilon_{U}\,{\left[\lambda \,\alpha_{U}+\left(\delta +\rho \right)\,\beta_{U}\right]}^{2}}{2\,\rho \,{\left(\delta +\rho \right)}^{2}\,{\left(1-\varepsilon_{U}\right)}^{2}\,\mu_{U}}-\frac{\gamma^{2}\,\pi_{L}^{2}\,\varepsilon_{L}\,{\left[\lambda \,\alpha_{L}+\left(\delta +\rho \right)\,\beta_{L}\right]}^{2}}{2\,\rho \,{\left(\delta +\rho \right)}^{2}\,{\left(1-\varepsilon_{L}\right)}^{2}\,\mu_{L}}$$

(87) $$j_{1}=\frac{\lambda \,\pi_{L}\,\gamma }{\delta +\rho }$$

$$j_{2}=\frac{\gamma \,\omega \,\pi_{U}\,\left[\lambda \,\alpha_{U}+\left(\delta +\rho \right)\,\beta_{U}\right]}{\rho \,\left(\delta +\rho \right)\,\left(1-\varepsilon_{U}\right)\,\mu_{U}}+$$

$$\frac{\gamma^{2}\,\pi_{G}\,\pi_{L}\,{\left[\lambda \,\alpha_{G}+\left(\delta +\rho \right)\,\beta_{G}\right]}^{2}}{\rho \,\mu_{G}\,{\left(\delta +\rho \right)}^{2}}+\frac{\gamma^{2}\,\pi_{U}\,\pi_{L}\,{\left[\lambda \,\alpha_{U}+\left(\delta +\rho \right)\,\beta_{U}\right]}^{2}}{\rho \,\left(1-\varepsilon_{U}\right)\,\mu_{U}\,{\left(\delta +\rho \right)}^{2}}$$

(88) $$+\frac{\pi_{L}\,f}{\rho }+\frac{\gamma^{2}\,\pi_{L}^{2}\,{\left[\lambda \,\alpha_{L}+\left(\delta +\rho \right)\,\beta_{L}\right]}^{2}}{2\,\rho \,{\left(\delta +\rho \right)}^{2}\,\left(1-\varepsilon_{L}\right)\,\mu_{L}}$$

(89) $$k_{1}=\frac{\lambda \,\pi_{U}\,\gamma }{\delta +\rho }$$

$$k_{2}=\frac{\gamma^{2}\,\pi_{U}^{2}\,{\left[\lambda \,\alpha_{U}+\left(\delta +\rho \right)\,\beta_{U}\right]}^{2}}{2\,\rho \,\left(1-\varepsilon_{U}\right)\,\mu_{U}\,{\left(\delta +\rho \right)}^{2}}+$$

$$\frac{\gamma^{2}\,\pi_{G}\,\pi_{U}\,{\left[\lambda \,\alpha_{G}+\left(\delta +\rho \right)\,\beta_{G}\right]}^{2}}{\mu_{G}\,\rho \,{\left(\delta +\rho \right)}^{2}}+$$

(90) $$\frac{\gamma^{2}\,\pi_{L}\,\pi_{U}\,{\left[\lambda \,\alpha_{L}+\left(\delta +\rho \right)\,\beta_{L}\right]}^{2}}{\rho \,\left(1-\varepsilon_{L}\right)\,\mu_{L}\,{\left(\delta +\rho \right)}^{2}}+\frac{f\,\pi_{U}}{\rho }$$

By substituting the values of i_1_, j_1_, and k_1_ into Equations 74, 75, the ratios of the cost-related subsidies provided by the government to opinion leaders and Internet users can be obtained from Equations 57, 58. The values of i_2_, j_2_, and k_2_ in the case of 2π_*G*_ > π_*L*_ and 2π_*G*_ > π_*U*_ can then be obtained by using Equations 57, 58 in Equations 86, 88, and 90:

$$i_{2}=\frac{\gamma^{2}\,{\left(\pi_{L}+2\,\pi_{G}\right)}^{2}\,{\left[\lambda \,\alpha_{L}+\left(\delta +\rho \right)\,\beta_{L}\right]}^{2}}{8\,\rho \,{\left(\delta +\rho \right)}^{2}\,\mu_{L}}+$$

$$\frac{\gamma^{2}\,\pi_{G}^{2}\,{\left[\lambda \,\alpha_{G}+\left(\delta +\rho \right)\,\beta_{G}\right]}^{2}}{2\,\rho \,{\left(\delta +\rho \right)}^{2}\,\mu_{G}}+$$

(91) $$\frac{f\,\pi_{G}}{\rho }+\frac{\gamma^{2}\,{\left(\pi_{U}+2\,\pi_{G}\right)}^{2}\,{\left[\lambda \,\alpha_{U}+\left(\delta +\rho \right)\,\beta_{U}\right]}^{2}}{8\,\rho \,{\left(\delta +\rho \right)}^{2}\,\mu_{U}}$$

$$j_{2}=\frac{\gamma^{2}\,\pi_{G}\,\pi_{L}\,{\left[\lambda \,\alpha_{G}+\left(\delta +\rho \right)\,\beta_{G}\right]}^{2}}{\rho \,{\left(\delta +\rho \right)}^{2}\,\mu_{G}}+\frac{f\,\pi_{L}}{\rho }+$$

$$\frac{\gamma^{2}\,\pi_{L}\,\left(2\,\pi_{G}+\pi_{L}\right)\,{\left[\lambda \,\alpha_{L}+\left(\delta +\rho \right)\,\beta_{L}\right]}^{2}}{4\,\rho \,{\left(\delta +\rho \right)}^{2}\,\mu_{L}}+$$

$$\frac{\omega \,\gamma \,\left(2\,\pi_{G}+\pi_{U}\right)\,\left[\lambda \,\alpha_{U}+\left(\delta +\rho \right)\,\beta_{U}\right]}{2\,\rho \,\left(\delta +\rho \right)\,\mu_{U}}+$$

(92) $$\frac{\gamma^{2}\,\pi_{L}\,\left(2\,\pi_{G}+\pi_{U}\right)\,{\left[\lambda \,\alpha_{U}+\left(\delta +\rho \right)\,\beta_{U}\right]}^{2}}{2\,\rho \,{\left(\delta +\rho \right)}^{2}\,\mu_{U}}$$

$$k_{2}=\frac{\gamma^{2}\,\pi_{G}\,\pi_{U}\,{\left[\lambda \,\alpha_{G}+\left(\delta +\rho \right)\,\beta_{G}\right]}^{2}}{\rho \,{\left(\delta +\rho \right)}^{2}\,\mu_{G}}+\frac{\pi_{U}\,f}{\rho }+$$

$$\frac{\gamma^{2}\,\pi_{U}\,\left(2\,\pi_{G}+\pi_{L}\right)\,{\left[\lambda \,\alpha_{L}+\left(\delta +\rho \right)\,\beta_{L}\right]}^{2}}{2\,\rho \,{\left(\delta +\rho \right)}^{2}\,\mu_{L}}+$$

(93) $$\frac{\gamma^{2}\,\pi_{U}\,\left(2\,\pi_{G}+\pi_{U}\right)\,{\left[\lambda \,\alpha_{U}+\left(\delta +\rho \right)\,\beta_{U}\right]}^{2}}{4\,\rho \,{\left(\delta +\rho \right)}^{2}\,\mu_{U}}$$

By substituting the values of i_1_, j_1_, k_1,_ ε_*L*_, and ε_*U*_ into Equations 69, 70, and 72, the optimal equilibrium strategies of the government, opinion leaders, and Internet users can be obtained from Equations 59–61. These optimal equilibrium strategies can be used in turn to obtain the optimal trajectory and steady-state value of the total amount of real information (Equation 62). By entering the values of i_1_, i_2_, j_1_, j_2_, k_1_, and k_2_ into Equations 79–81, we can get the optimal benefits for the government, opinion leaders, and Internet users from Equations 63–65 as well as the optimal benefits of the system to correct false information from Equation (66). Theorem 3 is thus proven.

#### Deduction 3

Theorem 3 shows that when the conditions 2π_*G*_ > π_*L*_ and 2π_*G*_ > π_*U*_ are not satisfied, the optimal equilibrium strategies (i.e., efforts) of the government, opinion leaders, and Internet users are not affected by one another. Consistent with the scenario of decentralized decision-making, the degree of effort of an entity is related only to its own relevant parameters. When the conditions 2π_*G*_ > π_*L*_ and 2π_*G*_ > π_*U*_ are satisfied, that is, in the case of cost-subsidized decision-making, opinion leaders and Internet users receive a certain ratio of subsidies. At this time, the optimal equilibrium strategy (i.e., effort) of each entity is also affected by these government subsidies. The optimal trajectory of the total amount of real information is then determined by the efforts of each entity. The steady-state value of the total amount of real information is positively correlated with the optimal benefits for the three entities as well as the benefit of the entire system to correct false information. This means that the greater the effort invested, the greater the steady-state value of the total amount of real information, and the greater the benefits to the entity and the benefits of the system. The specific relationships of impact are shown in Table 3.

**TABLE 3:** The effects of the parameters on the optimal equilibrium strategies of the government, opinion leaders, and Internet users under cost-subsidized decision-making.

	γ	π_*G*_	π_*L*_	π_*U*_	λ	α_*G*_	α_*L*_	α_*U*_	β_*G*_	β_*L*_	β_*U*_	μ_*G*_	μ_*L*_	μ_*U*_
E_*G*_^N^	+1	+1	0	0	+1	+1	0	0	+1	0	0	−1	0	0
E_*L*_^N^	+1	0	+1	0	+1	0	+1	0	0	+1	0	0	−1	0
E_*U*_^N^	+1	0	0	+1	+1	0	0	+1	0	0	+1	0	0	−1
ε_*L*_	0	+1	−1	0	0	0	0	0	0	0	0	0	0	0
ε_*U*_	0	+1	0	−1	0	0	0	0	0	0	0	0	0	0

+1 for positive impact, −1 for negative impact, and 0 for no impact.

## Analysis of the model

### Comparative analysis

Theorem 3 shows that in the case of cost-subsidized decision-making, the government will provide opinion leaders and Internet users with certain cost subsidies to satisfy2π_*G*_ > π_*L*_ and 2π_*G*_ > π_*U*_. Therefore, we analyze the model under the premise of2π_*G*_ > π_*L*_ and 2π_*G*_ > π_*U*_. We compared the optimal equilibrium strategy of each actor, the steady-state value of the total amount of real information, the optimal benefits to each actor, and the optimal benefits of the entire system to correct false information under different decision-making scenarios. The following inferences were made:

#### Deduction 1

When 2π_*G*_ > π_*L*_ and 2π_*G*_ > π_*U*_ are satisfied, the order of optimal strategies for the government under scenarios of decentralized, centralized, and subsidized decision-making is ${}_{E_{L}^{N^{*}}<E_{L}^{S^{*}}<E_{L}^{C^{*}}}$, that for opinion leaders under the scenarios of decentralized, centralized, and subsidized decision-making is${}_{E_{L}^{N^{*}}<E_{L}^{S^{*}}<E_{L}^{C^{*}}}$, and that for Internet users is${}_{E_{U}^{N^{*}}<E_{U}^{S^{*}}<E_{U}^{C^{*}}}$.

#### Verification 4

We also compared the optimal equilibrium strategies of the government (Equations 10, 40, and 59), opinion leaders (Equations 11, 41, and 60), and netizens (Equations 12, 42, and 61):


(94) $$\left\{ \begin{array}{c} E_{G}^{N^{*}}-E_{G}^{S^{*}}=0\\ E_{G}^{C^{*}}-E_{G}^{N^{*}}=\frac{\gamma \,\pi_{G}\,\left[\lambda \,\alpha_{G}+\beta_{G}\,\left(\delta +\rho \right)\right]}{\mu_{G}\,\left(\delta +\rho \right)}>0 \end{array}\right.$$

(95) $$\left\{ \begin{array}{c} E_{L}^{S^{*}}-E_{L}^{N^{*}}=\frac{\gamma \,\left(2\,\pi_{G}-\pi_{L}\right)\,\left[\beta_{L}\,\left(\delta +\rho \right)+\alpha_{L}\,\lambda \right]}{2\,\mu_{L}\,\left(\delta +\rho \right)}>0\\ E_{L}^{C^{*}}-E_{L}^{S^{*}}=\frac{\gamma \,\left(\pi_{L}+2\,\pi_{U}\right)\,\left[\beta_{L}\,\left(\delta +\rho \right)+\alpha_{L}\,\lambda \right]}{2\,\mu_{L}\,\left(\delta +\rho \right)}>0 \end{array}\right.$$

(96) $$\left\{ \begin{array}{c} E_{U}^{S^{*}}-E_{U}^{N^{*}}=\frac{\gamma \,\left(2\,\pi_{G}-\pi_{U}\right)\,\left[\beta_{U}\,\left(\delta +\rho \right)+\alpha_{U}\,\lambda \right]}{2\,\mu_{U}\,\left(\delta +\rho \right)}>0\\ E_{U}^{C^{*}}-E_{U}^{S^{*}}\\ =\frac{2\,\omega \,\left(\delta +\rho \right)+\gamma \,\left[\beta_{U}\,\left(\delta +\rho \right)+\alpha_{U}\,\lambda \right]\,\left(2\,\pi_{L}+\pi_{U}\right)}{2\,\mu_{U}\,\left(\delta +\rho \right)}>0 \end{array}\right.$$

Because all the parameters in Equations (94), (95), and (96) are non-negative,$E_{G}^{N^{*}}=E_{G}^{S^{*}}<E_{G}^{C^{*}}$, $E_{L}^{N^{*}}<E_{L}^{S^{*}}<E_{L}^{C^{*}}$, and $E_{U}^{N^{*}}<E_{U}^{S^{*}}<E_{U}^{C^{*}}$. This verifies deduction 1.

Deduction 1 shows that when the government’s marginal flow revenue exceeded the respective marginal flow revenues of opinion leaders and Internet users by two times, their optimal efforts were Pareto-optimal in the case of centralized decision-making. Unlike in the case of decentralized decision-making, the opinion leaders and Internet users received cost subsidies from the government in the case of cost-subsidized decision-making such that their optimal efforts achieved Pareto improvement. The optimal effort by the government was the same in the cases of both decentralized and cost-subsidized decision-making.

#### Deduction 2

When 2π_*G*_ > π_*L*_ and 2π_*G*_ > π_*U*_ were satisfied, the steady-state values of the total amount of real information under the decentralized, centralized, and cost-subsidized decision-making scenarios were_*R_S^N <R_S^S <R_S^C*_ .

#### Verification 5

We compared these steady-state values (Equations 13, 43, and 62) and obtained the following:


(97) $$\left\{ \begin{array}{c} \begin{array}{ccc} R_{S}^{C}-R_{S}^{S} & = & \frac{\alpha_{G}\,\gamma \,\left[\beta_{G}\,\left(\delta +\rho \right)+\alpha_{G}\,\lambda \right]\,\left(\pi_{L}+\pi_{U}\right)}{\delta \,\mu_{G}\,\left(\delta +\rho \right)}\\ & & +\frac{\alpha_{L}\,\gamma \,\left[\beta_{L}\,\left(\delta +\rho \right)+\alpha_{L}\,\lambda \right]\,\left(\pi_{L}+2\,\pi_{U}\right)}{2\,\delta \,\mu_{L}\,\left(\delta +\rho \right)}\\ & & +\frac{\begin{array}{c} 2\,\alpha_{U}\,\omega \,\left(\delta +\rho \right)+\alpha_{U}\,\gamma \\ \left[\beta_{U}\,\left(\delta +\rho \right)+\alpha_{U}\,\lambda \right]\,\left(2\,\pi_{L}+\pi_{U}\right) \end{array}}{2\,\delta \,\mu_{U}\,\left(\delta +\rho \right)} \end{array}\\ \begin{array}{ccc} R_{S}^{S}-R_{S}^{N} & = & \frac{\alpha_{L}\,\gamma \,\left(2\,\pi_{G}-\pi_{L}\right)\,\left[\beta_{L}\,\left(\delta +\rho \right)+\alpha_{L}\,\lambda \right]}{2\,\delta \,\mu_{L}\,\left(\delta +\rho \right)}\\ & & +\frac{\alpha_{U}\,\gamma \,\left(2\,\pi_{G}-\pi_{U}\right)\,\left[\beta_{U}\,\left(\delta +\rho \right)+\alpha_{U}\,\lambda \right]}{2\,\delta \,\mu_{U}\,\left(\delta +\rho \right)} \end{array} \end{array}\right.$$

Because all parameters in Equation 97 are non-negative,$R_{S}^{N}<R_{S}^{S}<R_{S}^{C}$. This verifies deduction 2.

Deduction 2 shows that when two times the revenue of marginal flow of the government exceeded the respective revenues of the marginal flow of the opinion leaders and Internet users in all three scenarios, the steady-state value of the total amount of real information was Pareto-optimal in the case of centralized decision-making. Unlike in decentralized decision-making, the steady-state value of the total amount of real information achieved Pareto improvement in the case of cost-subsidized decision-making.

#### Deduction 3

When 2π_*G*_ > π_*L*_ and 2π_*G*_ > π_*U*_ were satisfied, the order of the optimal benefits for the government under decentralized and subsidized decision-making was$V_{G}^{N^{*}}\,\left(R\right)<V_{G}^{S^{*}}\,\left(R\right)$, that for opinion leaders was$V_{L}^{N^{*}}\,\left(R\right)<V_{L}^{S^{*}}\,\left(R\right)$, and that for Internet users was$V_{U}^{N^{*}}\,\left(R\right)<V_{U}^{S^{*}}\,\left(R\right)$.

The order of the optimal benefits of the system to correct false information under decentralized, centralized, and subsidized decision-making was: ^VN*^(*R*) < ^VS*^(*R*) < ^VC*^ = (*R*).

#### Verification 6

In the case of centralized decision-making, the government, opinion leaders, and Internet users all made decisions to maximize the benefits of the system. Therefore, we compared only the optimal benefits of the three in the cases of decentralized and cost-subsidized decision-making. First, we compared the optimal benefits of the government (Equations 14, 63), opinion leaders (Equations 15, 64), and Internet users (Equations 16, 65):

$$V_{G}^{S^{*}}\,\left(R\right)-V_{G}^{N^{*}}\,\left(R\right)=\frac{\lambda \,\pi_{G}\,\gamma }{\delta +\rho }\,\left(R_{S}^{S}-R_{S}^{N}\right)+$$

$$\frac{\gamma^{2}\,{\left(\pi_{L}-2\,\pi_{G}\right)}^{2}\,{\left[\lambda \,\alpha_{L}+\left(\delta +\rho \right)\,\beta_{L}\right]}^{2}}{8\,\rho \,{\left(\delta +\rho \right)}^{2}\,\mu_{L}}+$$

(98) $$\frac{\gamma^{2}\,{\left(\pi_{U}-2\,\pi_{G}\right)}^{2}\,{\left[\lambda \,\alpha_{U}+\left(\delta +\rho \right)\,\beta_{U}\right]}^{2}}{8\,\rho \,{\left(\delta +\rho \right)}^{2}\,\mu_{U}}$$

$$V_{L}^{S^{*}}\,\left(R\right)-V_{L}^{N^{*}}\,\left(R\right)=\frac{\lambda \,\pi_{L}\,\gamma }{\delta +\rho }\,\left(R_{S}^{S}-R_{S}^{N}\right)+$$

$$\frac{\gamma^{2}\,\pi_{L}\,\left(2\,\pi_{G}-\pi_{L}\right)\,{\left[\lambda \,\alpha_{L}+\left(\delta +\rho \right)\,\beta_{L}\right]}^{2}}{4\,\rho \,{\left(\delta +\rho \right)}^{2}\,\mu_{L}}+$$

$$\frac{\omega \,\gamma \,\left(2\,\pi_{G}-\pi_{U}\right)\,\left[\lambda \,\alpha_{U}+\left(\delta +\rho \right)\,\beta_{U}\right]}{2\,\rho \,\left(\delta +\rho \right)\,\mu_{U}}+$$

(99) $$\frac{\gamma^{2}\,\pi_{L}\,\left(2\,\pi_{G}-\pi_{U}\right)\,{\left[\lambda \,\alpha_{U}+\left(\delta +\rho \right)\,\beta_{U}\right]}^{2}}{2\,\rho \,{\left(\delta +\rho \right)}^{2}\,\mu_{U}}$$

$$V_{U}^{S^{*}}\,\left(R\right)-V_{U}^{N^{*}}\,\left(R\right)=\frac{\lambda \,\pi_{U}\,\gamma }{\delta +\rho }\,\left(R_{S}^{S}-R_{S}^{N}\right)+$$

$$\frac{\gamma^{2}\,\pi_{U}\,\left(2\,\pi_{G}-\pi_{L}\right)\,{\left[\lambda \,\alpha_{L}+\left(\delta +\rho \right)\,\beta_{L}\right]}^{2}}{2\,\rho \,{\left(\delta +\rho \right)}^{2}\,\mu_{L}}+$$

(100) $$\frac{\gamma^{2}\,\pi_{U}\,\left(2\,\pi_{G}-\pi_{U}\right)\,{\left[\lambda \,\alpha_{U}+\left(\delta +\rho \right)\,\beta_{U}\right]}^{2}}{4\,\rho \,{\left(\delta +\rho \right)}^{2}\,\mu_{U}}$$

Deduction 2 shows that the steady-state value of the total amount of real information under the cost-subsidized decision-making scenario was greater than that under the decentralized decision-making scenario. As the parameters in Equations 98–100 are non-negative, we can get$V_{G}^{N^{*}}\,\left(R\right)<V_{G}^{S^{*}}\,\left(R\right)$, $V_{L}^{N^{*}}\,\left(R\right)<V_{L}^{S^{*}}\,\left(R\right)$, and $V_{U}^{N^{*}}\,\left(R\right)<V_{U}^{S^{*}}\,\left(R\right)$.

Second, we compared the optimal benefits of the system to correct false information (Equations 17, 44, and 66). Because the optimal benefit of the system is the sum of the optimal benefits of each actor in the system, and$V_{G}^{N^{*}}\,\left(R\right)<V_{G}^{S^{*}}\,\left(R\right)$, $V_{L}^{N^{*}}\,\left(R\right)<V_{L}^{S^{*}}\,\left(R\right)$, and $V_{U}^{N^{*}}\,\left(R\right)<V_{U}^{S^{*}}\,\left(R\right)$, then the optimal benefit of the system under the decentralized decision-making scenario must be smaller than that under the cost-subsidized decision-making scenario. Therefore, only the optimal benefits (Equations 44, 66) of the system under the centralized and cost-subsidized decision-making scenarios were compared:

$$V^{C^{*}}\,\left(R\right)-V^{S^{*}}\,\left(R\right)=\frac{\lambda \,\gamma \,\left(\pi_{G}+\pi_{L}+\pi_{U}\right)}{\delta +\rho }\,\left(R_{S}^{C}-R_{S}^{S}\right)+$$

$$\frac{\gamma^{2}\,{\left(\pi_{L}+\pi_{U}\right)}^{2}\,{\left[\lambda \,\alpha_{G}+\left(\delta +\rho \right)\,\beta_{G}\right]}^{2}}{2\,\rho \,{\left(\delta +\rho \right)}^{2}\,\mu_{G}}+$$

$$\frac{\gamma^{2}\,{\left(2\,\pi_{G}+\pi_{L}\right)}^{2}\,{\left[\lambda \,\alpha_{L}+\left(\delta +\rho \right)\,\beta_{L}\right]}^{2}}{8\,\rho \,{\left(\delta +\rho \right)}^{2}\,\mu_{L}}+$$

$$\frac{\omega^{2}+\gamma \,\omega \,\beta_{U}\,\left(2\,\pi_{L}+\pi_{U}\right)}{2\,\rho \,\mu_{U}}+$$

$$\frac{\gamma^{2}\,{\left(2\,\pi_{L}+\pi_{U}\right)}^{2}\,\left({\left[\lambda \,\alpha_{U}+\left(\delta +\rho \right)\,\beta_{U}\right]}^{2}\right)}{8\,\rho \,{\left(\delta +\rho \right)}^{2}\,\mu_{U}}+$$

(101) $$\frac{\left(2\,\pi_{L}+\pi_{U}\right)\,\gamma \,\lambda \,\omega \,\alpha_{U}}{2\,\rho \,\left(\delta +\rho \right)\,\mu_{U}}$$

Deduction 2 shows that the steady-state value of the total amount of real information under the cost-subsidized decision-making scenario was smaller than that under the centralized decision-making scenario. As all the parameters in Equation 101 are non-negative,^VN*^(*R*) < ^VS*^(*R*) < ^VC*^(*R*). Deduction 3 is thus proven.

Deduction 3 shows that when the revenue of the government’s marginal flow exceeded the revenue of opinion leaders’ and Internet users’ marginal flows in all three scenarios, the optimal benefit of the system to correct false information achieved Pareto optimality in the case of centralized decision-making. Unlike in decentralized decision-making, the optimal benefits of the system to correct false information attained Pareto improvement in the case of cost-subsidized decision-making. The optimal benefits for the government, opinion leaders, and Internet users were also higher in this scenario.

### Numerical simulation of equilibrium results of the game

The above theoretical analysis shows the decisions of the government, opinion leaders, and Internet users under different scenarios. The optimal effort, optimal benefit of each actor, total amount of real information, and optimal benefit of the system to correct false information were influenced by the parameters considered here. We used MATLAB 2017 to provide a more intuitive, simulation-based analysis of the impacts of different parameters on the results of the model. The results are as follows:


(102) $$\left\{ \begin{array}{c} \mu_{G}=3,\mu_{L}=1.7,\mu_{U}=1.2,\alpha_{G}=2.5,\\ \alpha_{L}=1.8,\alpha_{U}=1.3,\delta =1,\\ R_{0}=0,f=1,\gamma =0.6,\beta_{G}=1.8,\beta_{L}=1.4,\beta_{U}=1.1,\\ \rho =0.2,\pi_{G}=3,\pi_{L}=2.4,\pi_{U}=1.8,\omega =1.3,\lambda =0.7 \end{array}\right.$$

We obtained the equilibrium results of the differential game model under the three scenarios of decentralized, centralized, and cost-subsidized decision-making based on the results of the above assignments (Equation 102), as shown in Table 4.

**TABLE 4:** Equilibrium results of the differential game model in scenarios of decentralized, centralized, and cost-subsidized decision-making.

	Decentralized decision	Centralized decision	Cost-subsidized decision
E_*G*_	1.8300	4.3920	1.8300
E_*L*_	1.9482	5.8447	3.4094
E_*U*_	1.5750	7.3833	3.4125
ε_*L*_	−	−	0.4286
ε_*U*_	−	−	0.5385
R_*S*_	10.1293	31.0988	15.1482
V_*G*_	114.3679	−	138.0880
V_*L*_	105.6938	−	156.5022
V_*U*_	76.2489	−	105.7891
V_*S*_	296.3106	556.5692	400.3792

The results in Table 4 verify the correctness of the theoretical analysis of deductions 1–3 above.

**FIGURE 1:**
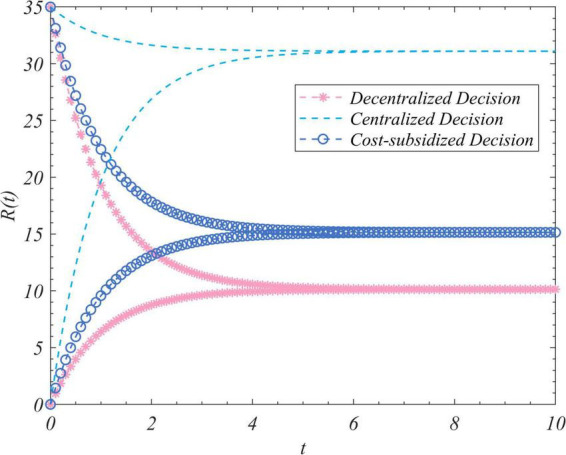
The trajectories of the evolution of the total amount of real information in the cases of decentralized, centralized, and cost-subsidized decision-making when R_0_ = 0 and 35.

**FIGURE 2:**
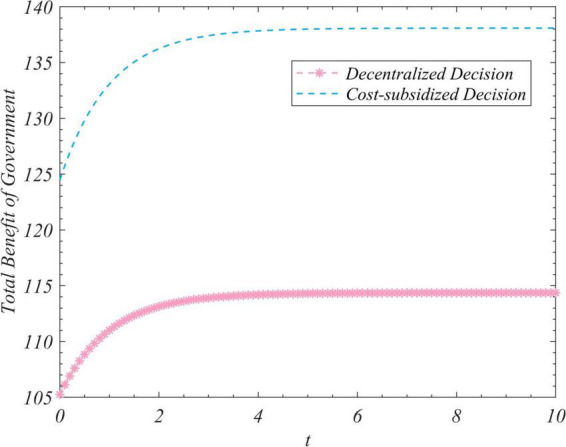
The trends of the gross revenue of the government under the decentralized and subsidized decision-making scenarios.

**FIGURE 3:**
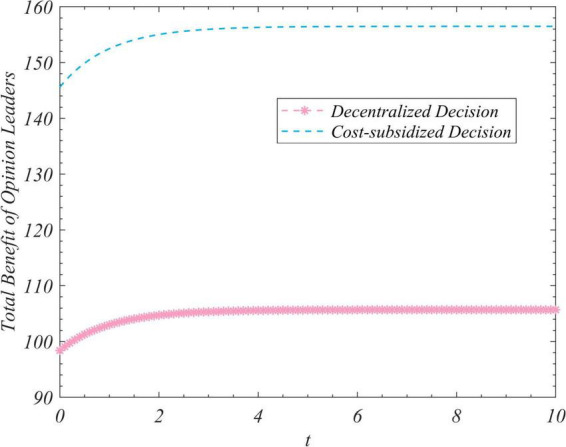
Trends of the total income of opinion leaders under the decentralized and subsidized decision-making scenarios.

**FIGURE 4:**
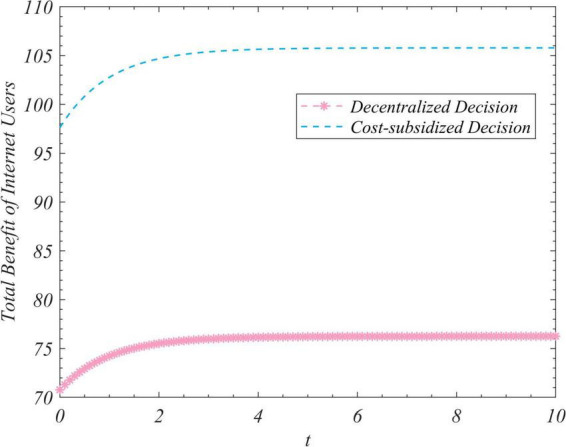
Trends of the total revenue of Internet users under the decentralized and subsidized decision-making scenarios.

**FIGURE 5:**
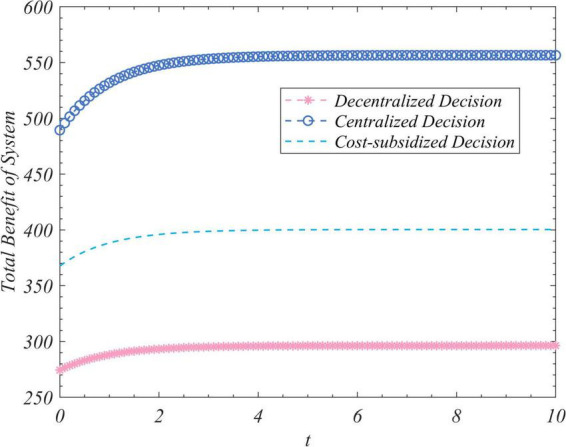
Trends of the total revenue of the system to correct false information under the decentralized, centralized, and subsidized decision-making scenarios.

We used the results (Equation 102) to conduct simulations to analyze the trajectory of change in the total amount of real information, the total incomes of the three actors, and the total income of the system to correct false information under the different decision-making scenarios considered here. We set R_0_ to zero and 35 and ran the program two times while keeping the values of the other parameters unchanged. Figure 1 depicts the time-evolution trajectories of the total amount of real information when the initial values were zero and 35. The trends of the total incomes over time of the government, opinion leaders, and Internet users are shown in Figures 2–4, respectively. The trend of income of the system to correct false information over time is shown in Figure 5.

Figure 1 shows that regardless of the initial value of the total amount of real information on the social platform, it eventually converged to the same steady-state value over time. This shows that the steady-state value of the total amount of real information was independent of its initial value, and was related only to the decision-making scenario. This value was the largest in the case of centralized decision-making, followed by cost-subsidized and decentralized decision-making. Figures 2–4 show that regardless of whether decentralized or subsidized decision-making was considered, the total revenues of the government, opinion leaders, and Internet users increased over time but did not grow further after reaching a certain value. The total revenue of the three actors under the cost-subsidized decision-making scenario was greater than that under the decentralized scenario. However, the government needed to bear the costs of additional subsidies in the former scenario. However, because of the total amount of real information attained by Pareto improvement, the government’s income from social platforms also achieved Pareto improvement. Figure 5 shows that the total revenue of the system to correct false information increased over time regardless of the decision-making scenario considered, but did not increase after reaching a certain value. This revenue was the largest under the centralized decision-making scenario, followed by the cost-subsidized and decentralized decision-making scenarios.

### Sensitivity analysis of related parameters

We conducted a sensitivity analysis of the important parameters in our model. Diagrams of the sensitivity analysis of only some parameters are provided below. The remaining parameters were subjected to a sensitivity analysis ranging from small to large according to reference values of −20, −10, +10, and +20%. The influence of the changes in the parameter values on the equilibrium results of the model was analyzed. The results are shown in Table 5.

**TABLE 5:** Sensitivity analysis of each parameter in the model.

Parameter	$R_{S}^{N}$	^VN*^(*R*)	$R_{S}^{C}$	^VC*^(*R*)	$R_{S}^{S}$	^VS*^(*R*)
λ = (0.56→0.84)	+1	+1	+1	+1	+1	+1
ω = (1.04→1.56)	0	+1	+1	+1	0	+1
γ = (0.48→0.72)	+1	+1	+1	+1	+1	+1
*f* = (0.8→1.2)	0	+1	0	+1	0	+1
μ_*G*_ = (2.4→3.6)	−1	−1	−1	−1	−1	−1
μ_*L*_ = (1.36→2.04)	−1	−1	−1	−1	−1	−1
μ_*U*_ = (0.96→1.44)	−1	−1	−1	−1	−1	−1
π_*G*_ = (2.4→3.6)	+1	+1	+1	+1	+1	+1
π_*L*_ = (1.92→2.88)	+1	+1	+1	+1	+1	+1
π_*U*_ = (1.504→2.256)	+1	+1	+1	+1	+1	+1
α_*G*_ = (2→3)	+1	+1	+1	+1	+1	+1
α_*L*_ = (1.44→2.16)	+1	+1	+1	+1	+1	+1
α_*U*_ = (1.04→1.56)	+1	+1	+1	+1	+1	+1
β_*G*_ = (1.44→2.16)	+1	+1	+1	+1	+1	+1
β_*L*_ = (1.12→1.68)	+1	+1	+1	+1	+1	+1
β_*U*_ = (0.88→1.32)	+1	+1	+1	+1	+1	+1
δ = (0.8→1.2)	−1	−1	−1	−1	−1	−1
ρ = (0.16→0.24)	−1	−1	−1	−1	−1	−1

+1 indicates positive correlation, −1 indicates negative correlation, and 0 indicates no correlation.

**FIGURE 6:**
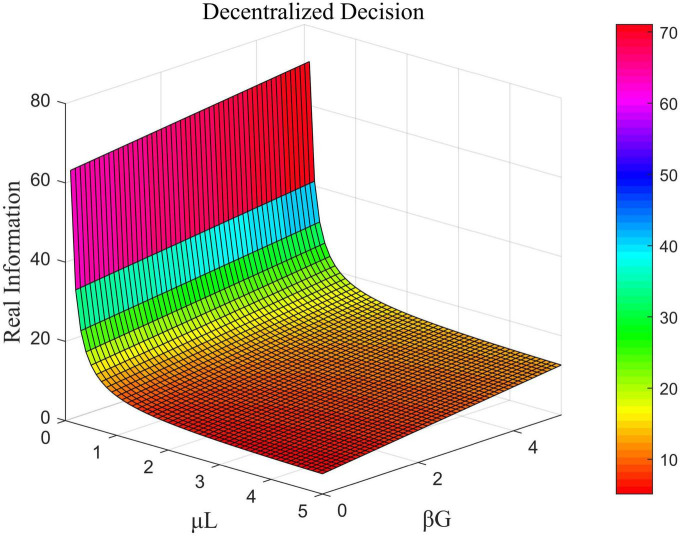
Effects of μ_*L*_ and β_*G*_ on the total amount of real information under decentralized decision-making.

**FIGURE 7:**
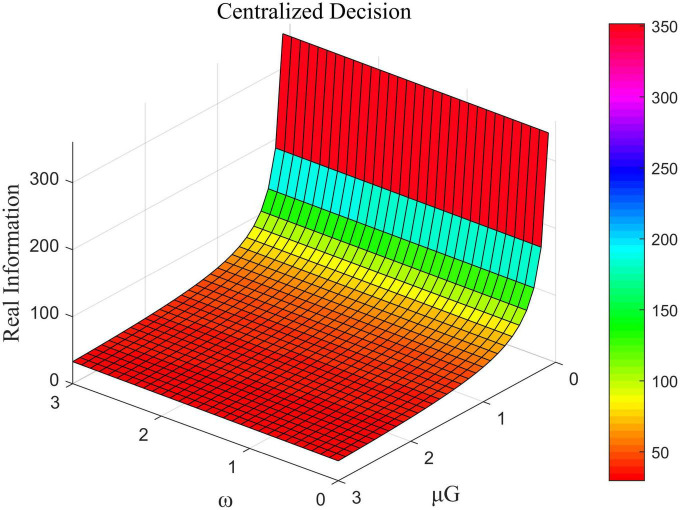
The influence of ω and μ_*G*_ on the total amount of real information under centralized decision-making.

**FIGURE 8:**
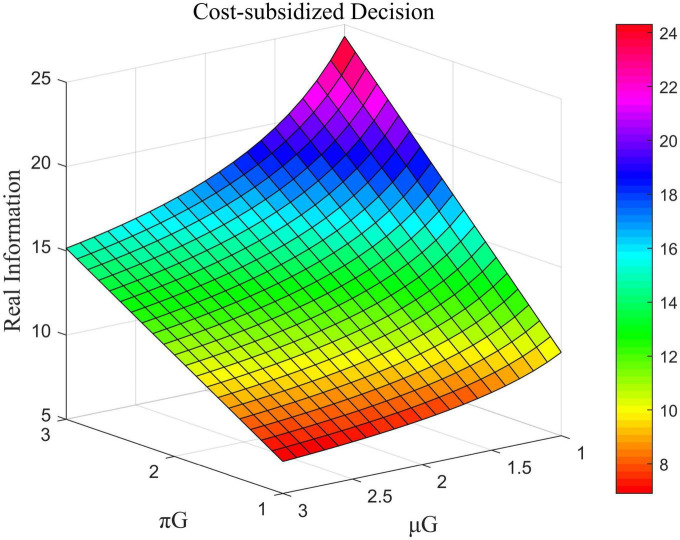
Impact of π_*G*_ and μ_*G*_ on the total amount of real information under cost-subsidized decision-making.

The influence of μ_*L*_ and β_*G*_ on the total amount of real information under decentralized decision-making, that of ω and μ_*G*_ on the total amount of real information under centralized decision-making, and the impact of μ_*U*_ and μ_*G*_ on the total amount of real information under cost-subsidized decision-making is shown in Figures 6–8, respectively.

Figure 6 shows that in the case of decentralized decision-making, the coefficient of the cost of efforts by opinion leaders was negatively correlated with the total amount of real information while that of the government was positively correlated with it. Figure 7 shows that in the case of centralized decision-making, the direct interests of opinion leaders and Internet users had no impact on the total amount of real information. This means that the value of ω did not influence the amount of real information. The coefficient of the cost of government effort was negatively correlated with the total amount of real information in this case. Figure 8 shows that under the cost-subsidized decision-making scenario, the revenue of marginal flow of the government was positively correlated with the total amount of real information, while the coefficient of the cost of government effort was negatively correlated with it.

The results in Table 5 show the following:

(1) Regardless of the decision-making scenario considered, the degree of attention accorded after the occurrence of major emergencies, the coefficients of influence of the government, opinion leaders, and Internet users on social platforms, their marginal flow incomes, the coefficient of influence of their own efforts on the total amount of real information, and the coefficient of influence of the total amount of real information on its flow had a positive impact on the equilibrium results of the model.
(2) Regardless of the decision-making scenario considered, the coefficients of the cost of effort, subsidies, and the coefficients of the dissemination of real information of the government, opinion leaders, and Internet users had a negative impact on the equilibrium results of the model.
(3) Regardless of the decision-making scenario considered, the initial traffic on social platforms had a positive impact on the total revenue of the system to correct misinformation. However, it had no effect on the total amount of real information. The direct interests of opinion leaders and Internet users had a positive impact on the total income of the system to correct false information in any decision-making scenario, but only in centralized decision-making situations did they have a positive impact on the total amount of real information and had no impact on it in the other two decision-making scenarios considered.

## Conclusion

A large amount of false information related to major incidents appears and spreads quickly on social networking platforms soon after their occurrence. This can cause public panic and threaten social stability. In this context, this article regarded the government, opinion leaders, and Internet users as part of a system to correct false information. We used optimal control theory and differential game theory to formulate a differential game model under three decision-making scenarios: decentralized, centralized, and cost-subsidized. By solving for the optimal equilibrium strategy of each actor in the model under the different decision-making situations, we obtained their respective optimal benefits, the optimal trajectory of the total amount of real information in the system, and the optimal benefits of the system to correct false information. The following conclusions were obtained:

(1) In the case of decentralized decision-making, the optimal equilibrium strategies of the government, opinion leaders, and Internet users, their respective optimal benefits, the optimal trajectory of the total amount of real information, and the optimal benefits of the system to correct false information were the lowest. This is because all three actors made decisions to maximize their own interests in this case and did not consider the interests of one another. Such decision-making situations are most likely to occur in practice, and minimizing the adverse effects of false information is challenging in such cases.
(2) In centralized decision-making situations, each actor made decisions to maximize the benefit of the system. Although this increased the cost of their own effort, the overall system achieved global Pareto optimality. However, opinion leaders and Internet users are bound by their own benefits in the real world. Therefore, centralized decision-making is only an unrepresentative theoretical possibility.
(3) In the case of cost-subsidized decision-making, the government provided certain subsidies to opinion leaders and Internet users to motivate them to participate in correcting false information. This reduced the cost of their efforts. The equilibrium results of the model, in this case, did not reach global Pareto optimality. However, they attained a certain degree of Pareto improvement compared with decentralized decision-making. This kind of decision-making scenario is the most valuable reference for practical use. Because the government is responsible for maintaining social stability, it should provide appropriate cost subsidies to opinion leaders and Internet users in order to reduce public panic by combating online misinformation.

## Data availability statement

The original contributions presented in this study are included in the article/supplementary material, further inquiries can be directed to the corresponding author.

## Author contributions

BL and HL contributed to the conceptualization. BL contributed to the methodology, software, and writing—original draft preparation. BL and RL contributed to the validation. HL and QS contributed to the writing—review and editing and funding acquisition. All authors have read and agreed to the published version of the manuscript.

## References

[B1] Agarwal P. Al Aziz R. Zhuang J. (2022). Interplay of rumor propagation and clarification on social media during crisis events-A game-theoretic approach. *Eur. J. Oper. Res.* 298 714–733. 10.1016/j.ejor.2021.06.060

[B2] Akalu T. Y. Gelaye K. A. Bishaw M. A. Tilahun S. Y. Yeshaw Y. Azale T. (2021). Depression, Anxiety, and stress symptoms and its associated factors among residents of gondar town during the early Stage of COVID-19 pandemic. *Risk Manag. Healthc. Policy* 14 1073–1083. 10.2147/RMHP.S296796 33758560 PMC7979341

[B3] Aleahmad A. Karisani P. Rahgozar M. Oroumchian F. (2016). OLFinder: Finding opinion leaders in online social networks. *J. Inf. Sci.* 42 659–674. 10.1177/0165551515605217

[B4] Bamakan S. M. H. Nurgaliev I. Qu Q. (2019). Opinion leader detection: A methodological review. *Expert Syst. Appl.* 115 200–222. 10.1016/j.eswa.2018.07.069

[B5] Biancardi M. Iannucci G. Villani G. (2022). Groundwater Exploitation and illegal behaviors in a differential game. *Dyn. Games Appl.* 12 996–1009. 10.1007/s13235-022-00436-0

[B6] Bordia P. DiFonzo N. Haines R. Chaseling E. (2005). Rumors denials as persuasive messages: Effects of personal relevance, source, and message characteristics. *J. Appl. Soc. Psychol.* 35 1301–1331. 10.1111/j.1559-1816.2005.tb02172.x

[B7] Bouanan Y. Zacharewicz G. Vallespir B. (2016). DEVS modelling and simulation of human social interaction and influence. *Eng. Appl. Artif. Intell.* 50 83–92.

[B8] Buchanan T. Benson V. (2019). Spreading Disinformation on facebook: Do Trust in message source, risk propensity, or personality affect the organic reach of “Fake News”? *Soc. Media Soc.* 5 1–9. 10.1177/2056305119888654

[B9] Cao J. Xu X. Chen X. (2019). Risk evolution model for large group emergency decision-making influenced by extreme preference. *Syst. Eng. Theory Pract.* 39 596–614.

[B10] Din A. U. Han H. Ariza-Montes A. Vega-Munoz A. Raposo A. Mohapatra S. (2022). The Impact of COVID-19 on the Food supply chain and the role of E-Commerce for Food Purchasing. *Sustainability* 14:3074. 10.3390/su14053074

[B11] Fibich G. Gavious A. Lowengart O. (2003). Explicit solutions of optimization models and differential games with nonsmooth (asymmetric) reference-price effects. *Oper. Res.* 51 721–734. 10.1287/opre.51.5.721.16758 19642375

[B12] Garcia-Meza M. A. (2021). The cost of work discrimination: A Market capture differential game model. *Mathematics* 9:2419. 10.3390/math9192419

[B13] Guess A. Nagler J. Tucker J. (2019). Less than you think: Prevalence and predictors of fake news dissemination on Facebook. *Sci. Adv.* 5:eaau4586. 10.1126/sciadv.aau4586 30662946 PMC6326755

[B14] Hong X. Zhang G. J. Lu D. J. Liu H. Zhu L. Xu M. L. (2022). Personalized Crowd emotional contagion coupling the virtual and physical cyberspace. *IEEE Trans. Syst. Man Cybernet. Syst.* 52 1638–1652. 10.1109/TSMC.2020.3034395

[B15] Hosseini S. Zandvakili A. (2022). Information dissemination modeling based on rumor propagation in online social networks with fuzzy logic. *Soc. Netw. Anal. Mining* 12:34. 10.1007/s13278-022-00859-y

[B16] Jain L. Katarya R. (2019). Discover opinion leader in online social network using firefly algorithm. *Expert Syst. Appl.* 122 1–15. 10.1016/j.eswa.2018.12.043

[B17] Kang J. Zeng Y. Chen S. Wang Y. (2022). E-commerce platform response to major public emergencies-Optimal strategies and benefits of e-commerce platform subsidies. *Syst. Eng. Theory Pract.* 42 345–367.

[B18] Li B. Li H. Sun Q. Lv R. Zhao J. (2022). Evolutionary Game analysis of the dissemination of false information by multiple parties after major emergencies. *Complexity* 2022:3527674. 10.1155/2022/3527674

[B19] Liao H. P. Wang J. L. (2021). The impact of epidemic information on the public’s worries and attitude toward epidemic prevention measures during the COVID-19 outbreak. *Curr. Psychol.* 10.1007/s12144-021-01364-9 [Epub ahead of print]. 33531791 PMC7842393

[B20] Liu H. (2022). Official social media and its impact on public behavior during the first wave of COVID-19 in China. *BMC Public Health* 22:428. 10.1186/s12889-022-12803-y 35241057 PMC8893355

[B21] Machowska D. Nowakowski A. Wiszniewska-Matyszkiel A. (2022). Closed-loop Nash equilibrium for a partial differential game with application to competitive personalized advertising. *Automatica* 140. 10.1016/j.automatica.2022.110220

[B22] McElroy E. Patalay P. Moltrecht B. Shevlin M. Shum A. Creswell C. (2020). Demographic and health factors associated with pandemic anxiety in the context of COVID-19. *Br. J. Health Psychol.* 25 934–944. 10.1111/bjhp.12470 32860334

[B23] Ozturk P. Li H. Y. Sakamoto Y. (2015). “Combating rumor spread on social media: The Effectiveness of Refutation and Warning,” in *Proceedings of the 2015 48th Hawaii international conference on system sciences (Hicss)*, Kauai, HI.

[B24] Pal A. Banerjee S. (2021). Internet users beware, you follow online health rumors (more than counter-rumors) irrespective of risk propensity and prior endorsement. *Inf. Technol. People* 34 1721–1739. 10.1108/ITP-02-2019-0097

[B25] Perelman A. Shima T. Rusnak I. (2011). Cooperative differential games strategies for active aircraft protection from a homing missile. *J. Guid. Control Dyn.* 34 761–773. 10.2514/1.51611

[B26] Prasad A. Sethi S. P. (2004). Competitive advertising under uncertainty: A stochastic differential game approach. *J. Optim. Theory Appl.* 123 163–185. 10.1023/B:JOTA.0000043996.62867.20

[B27] Shchelchkov K. A. (2022). Estimate of the Capture time and construction of the Pursuer’s Strategy in a nonlinear two-person differential game. *Differ. Equ.* 58 264–274. 10.1134/S0012266122020112

[B28] Vosoughi S. Roy D. Aral S. (2018). The spread of true and false news online. *Science* 359:1146. 10.1126/science.aap9559 29590045

[B29] Wu X. Liu X. Zhou J. (2019). Evolution model of NIMBY opinion based on public perception and governmental guidance. *Syst. Eng. Theory Pract.* 39 2865–2879.

[B30] Xu Z. Cheng Y. Yao S. (2021). Tripartite evolutionary game model for public health emergencies. *Discrete Dyn. Nat. Soc.* 2021:6693597. 10.1155/2021/6693597

[B31] Yu Z. Lu S. Wang D. Li Z. (2021). Modeling and analysis of rumor propagation in social networks. *Information Sci.* 580 857–873.

[B32] Zhang H. G. Wei Q. L. Liu D. R. (2011). An iterative adaptive dynamic programming method for solving a class of nonlinear zero-sum differential games. *Automatica* 47 207–214. 10.1016/j.automatica.2010.10.033

